# Histological and transcriptomic analysis of muscular atrophy associated with depleted flesh pigmentation in Atlantic salmon (*Salmo salar*) exposed to elevated seawater temperatures

**DOI:** 10.1038/s41598-023-31242-2

**Published:** 2023-03-14

**Authors:** Thu Thi Minh Vo, Gianluca Amoroso, Tomer Ventura, Abigail Elizur

**Affiliations:** 1 Centre for Bioinnovation, University of the Sunshine Coast, 4 Locked Bag, Maroochydore DC, QLD 4558 Australia; 2 School of Science, Technology and Engineering, University of the Sunshine Coast, 4 Locked Bag, Maroochydore DC, QLD 4558 Australia; 3 Institute for Marine and Antarctic Studies, University of Tasmania, Private Bag 49, Hobart, TAS 7001 Australia; 4 School of Biotechnology, International University, Vietnam National University, Ho Chi Minh City, 700000 Vietnam

**Keywords:** Transcriptomics, Feeding behaviour

## Abstract

Tasmania is experiencing increasing seawater temperatures during the summer period which often leads to thermal stress-induced starvation events in farmed Atlantic salmon, with consequent flesh pigment depletion. Our previous transcriptomic studies found a link between flesh pigmentation and the expression of genes regulating lipid metabolism accompanied by feeding behavior in the hindgut. However, the impact of prolonged exposure to elevated water temperature on muscle structural integrity and molecular mechanisms in muscle underlying pigment variation has not been elucidated to date. In this study, we investigated the effect of prolonged exposure to elevated water temperature on the farmed salmon flesh pigmentation and structural integrity, using muscle histological and transcriptomic analysis. On April 2019, after the end of the summer, two muscle regions of the fish fillet, front dorsal and back central (usually the most and least affected by depletion, respectively), were sampled from fifteen fish (weighing approximately 2 kg and belonging to the same commercial population split in two cages). The fish represented three flesh color intensity groups (n = 5 fish per group) categorized according to general level of pigmentation and presence of banding (i.e. difference in color between the two regions of interest) as follows: high red color-no banding (HN), high red color-banded (HB) and Pale fish. Histological analysis showed a distinction between the flesh color intensity phenotypes in both muscle regions. Muscle fibers in the HB fish were partly degraded, while they were atrophied and smaller in size in Pale fish compared to HN fish. In the Pale fish, interstitial spaces between muscle fibers were also enlarged. Transcriptomic analysis showed that in the front dorsal region of the HN fish, genes encoding collagens, calcium ion binding and metabolic processes were upregulated while genes related to lipid and fatty acid metabolism were downregulated when compared to HB fish. When comparing the back central region of the three phenotypes, actin alpha skeletal muscle and myosin genes were upregulated in the HN and HB fish, while tropomyosin genes were upregulated in the Pale fish. Also, genes encoding heat shock proteins were upregulated in the HN fish, while genes involving lipid metabolism and proteolysis were upregulated in the Pale fish. Starvation, likely caused by thermal stress during prolonged periods of elevated summer water temperatures, negatively affects energy metabolism to different extents, leading to localized or almost complete flesh color depletion in farmed Atlantic salmon. Based on our results, we conclude that thermal stress is responsible not only for flesh discoloration but also for loss of muscle integrity, which likely plays a key role in pigment depletion.

## Introduction

Tasmania is increasingly impacted by climate change and is ahead of global trends^1,2^, which poses a risk to the Atlantic salmon industry, urging additional research on thermal tolerance and linked quality traits to ensure both performance and welfare. In recent years, prolonged periods of elevated seawater temperatures (over 20 °C) during summer in Tasmania have exceeded the Atlantic salmon optimal temperature range of 12–18 °C^3,4^, resulting in thermal stress-induced starvation and localized or complete flesh pigment depletion^4–10^. In wild salmonids, flesh pigmentation with its unique pink-red color is formed by the deposition of carotenoids, primarily astaxanthin, derived from the ingestion of algae, and/or crustaceans feeding on algae^11^. In farmed salmonids, a synthesized form of dietary carotenoids is added to commercial feeds to obtain the desired flesh pigmentation^11,12^. The level at which carotenoids are supplemented and the resulting flesh pigmentation affect product marketability, with the inclusion of carotenoids in commercial feeds representing a considerable cost for the producers^13,14^.

During a period of thermal stress in Tasmania, our recent research found that salmon flesh pigmentation was reduced/impacted to different extents within the same group: some individuals maintained red color intensity (high red color-no banding); some fish were discolored at the front dorsal region (high red color-banded), while others were almost entirely depleted of flesh color (Pale)^10^. Also, Wade, et al.^5^ observed the same phenomenon and found a strong positive correlation between fillet pigmentation and fish body condition. The authors also observed that loss of pigmentation was accompanied by voluntary cessation of feed intake and impaired osmoregulation with compromised liver and renal functions. The same pattern of pigment depletion was observed also under experimental conditions and at a smaller fish size after Atlantic salmon were exposed to a period of elevated temperature and forced starvation^6,7^. This confirms a generalized deleterious effect of elevated temperature on flesh pigmentation in Atlantic salmon.

Our previous studies on Atlantic salmon identified strong correlations between flesh pigmentation and the pyloric caeca and the gut microbiota^8,10^. Using gut transcriptome analysis, we found that flesh pigmentation also correlates with the expression of genes related to lipid metabolism, feeding behavior, carotenoid-binding cholesterol and oxidative stress. Also, we demonstrated the presence of a genetic effect on flesh pigment depletion in Atlantic salmon, suggesting that selection for thermal-tolerant families with improved flesh quality could benefit the Tasmanian Atlantic salmon farming industry^4,9^.

In salmonids muscle, astaxanthin is bound to the actomyosin complex^15–17^ and F-actin rather than myosin^18^. A positive relationship between muscle fiber density, flesh quality, pigment content and red color intensity was found in Atlantic salmon^19–21^. In fish, genetics and environmental conditions can significantly affect muscle fiber density^22–25^, and the distribution of muscle fiber associates with a broad-range of structural (connective tissue including endomysium, perimysium and epimysium, cytoskeletal and contractile proteins) and metabolic components^26,27^. Moreover, texture characteristics and color visualization significantly correlate with muscle fiber density^22^. At the gene expression level, the upregulation of genes involved in proteolysis, aerobic metabolism and collagenase proteolytic pathways was connected with muscle deterioration in rainbow trout^28^. Also, several genes involved in actin remodeling and glucose homeostasis were upregulated in Atlantic salmon fed astaxanthin-supplemented diet^29^. Research so far has focused primarily on defining phenotypic correlations with feed intake or on manipulating type and inclusion rate of pigments in the feed. Nevertheless, the mechanisms behind pigment loss in salmon flesh still remain elusive in this species. In addition to that, our understanding of how elevated water temperatures in Tasmania impact farmed Atlantic salmon flesh quality and whether flesh pigmentation correlates with muscle structural integrity remains limited, yet relevant to both farmed and wild salmon populations. Thus, the objective of this study was to investigate the effect of prolonged exposure to elevated seawater temperatures during summer in Tasmania on the flesh quality of farmed Atlantic salmon, addressing muscle pigmentation, muscle structural integrity and the link between the two.

## Results

**Figure 1:**
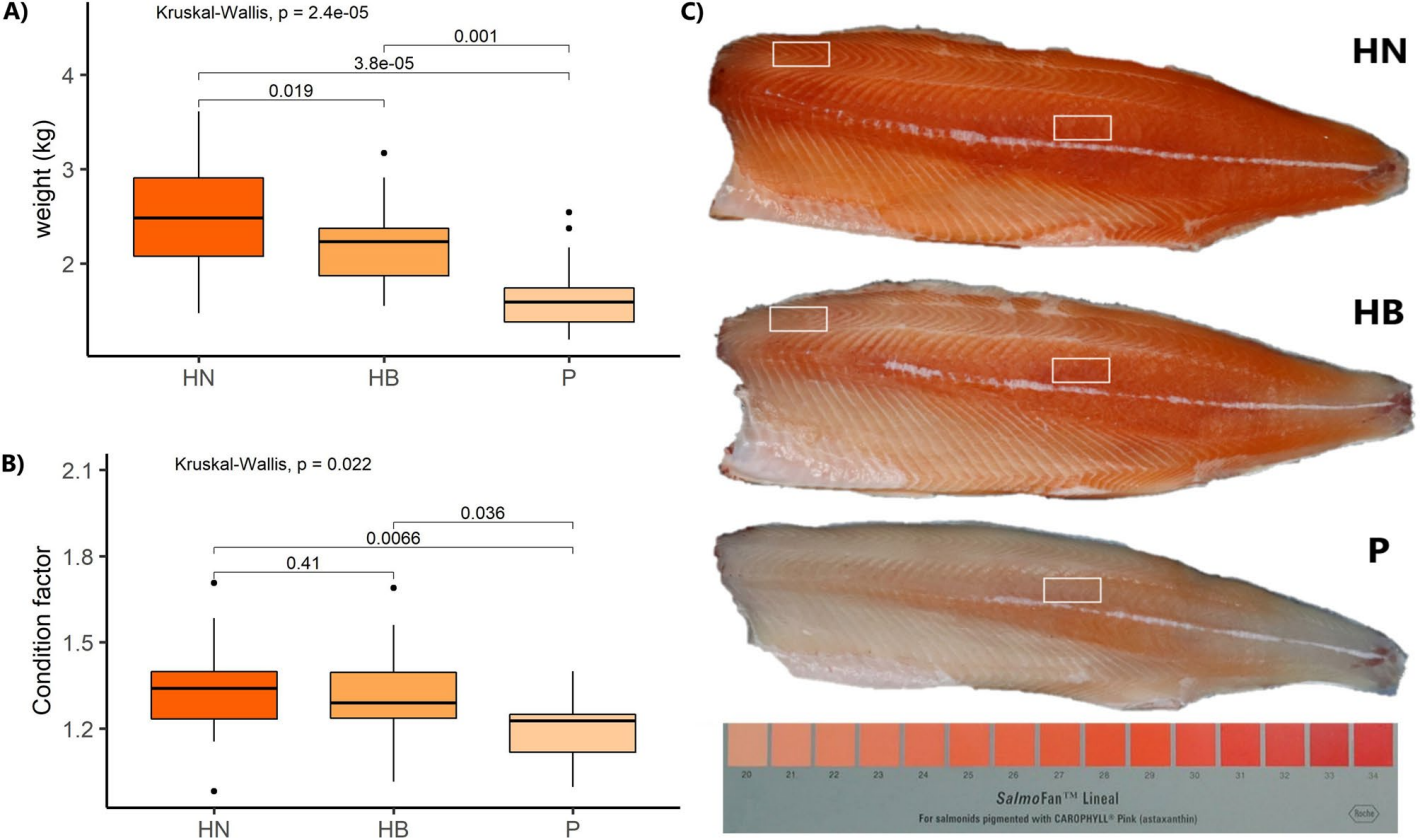
Weight (**A**) and condition factor (**B**) of fish associates with flesh phenotype. HN: High red color-no banding, HB: High red color-banded, P: pale (n = 39 for HN, n = 25 for HB fish and n = 13 for pale fish). Significant differences were determined by one-way ANOVA (*P* ≤ 0.05). (**C**) The grouping of experimental fish based on flesh color. FD (front dorsal) and BC (back central) regions sampled are marked by white squares. The classification of flesh color phenotypes is based on a score measured using SalmoFan™ Lineal in the front dorsal and back central regions: High red color-no banding (HN) fish, High red color-banded (HB) fish, Pale (P) fish.

Following the flesh pigment classification, there were 39 HN fish, 25 HB fish, and 13 Pale fish. Their average weights were 2.503 ± 0.539, 2.192 ± 0.373 and 1.682 ± 0.422 kg (mean ± SD), and their condition factors were 1.34 ± 0.14, 1.31 ± 0.14 and 1.20 ± 0.12 (mean ± SD) for HN, HB and Pale phenotype, respectively (Fig. 1). The weights of the three groups are significantly different (*P* < 0.05). The condition factor of Pale fish was significantly different from HN fish (*P* < 0.01) and HB fish (*P* < 0.05), while there was no significant difference between HN and HB fish (*P* > 0.05). The average weight and condition factor of the fifteen fish (n = 5 per group) chosen from the above 77 fish as representative were 2.578 ± 0.402, 2.124 ± 0.327, and 1.448 ± 0.133 and 1.37 ± 0.20, 1.27 ± 0.08 and 1.17 ± 0.16 for HN, HB and Pale fish, respectively. In the representative groups, the weight and condition factor of Pale fish was significantly different from HN (*P* < 0.05). There was no significant difference in other comparisons *(P* > 0.05).

### Histological analysis of two muscle regions shows changes between flesh color phenotypes

**Figure 2:**
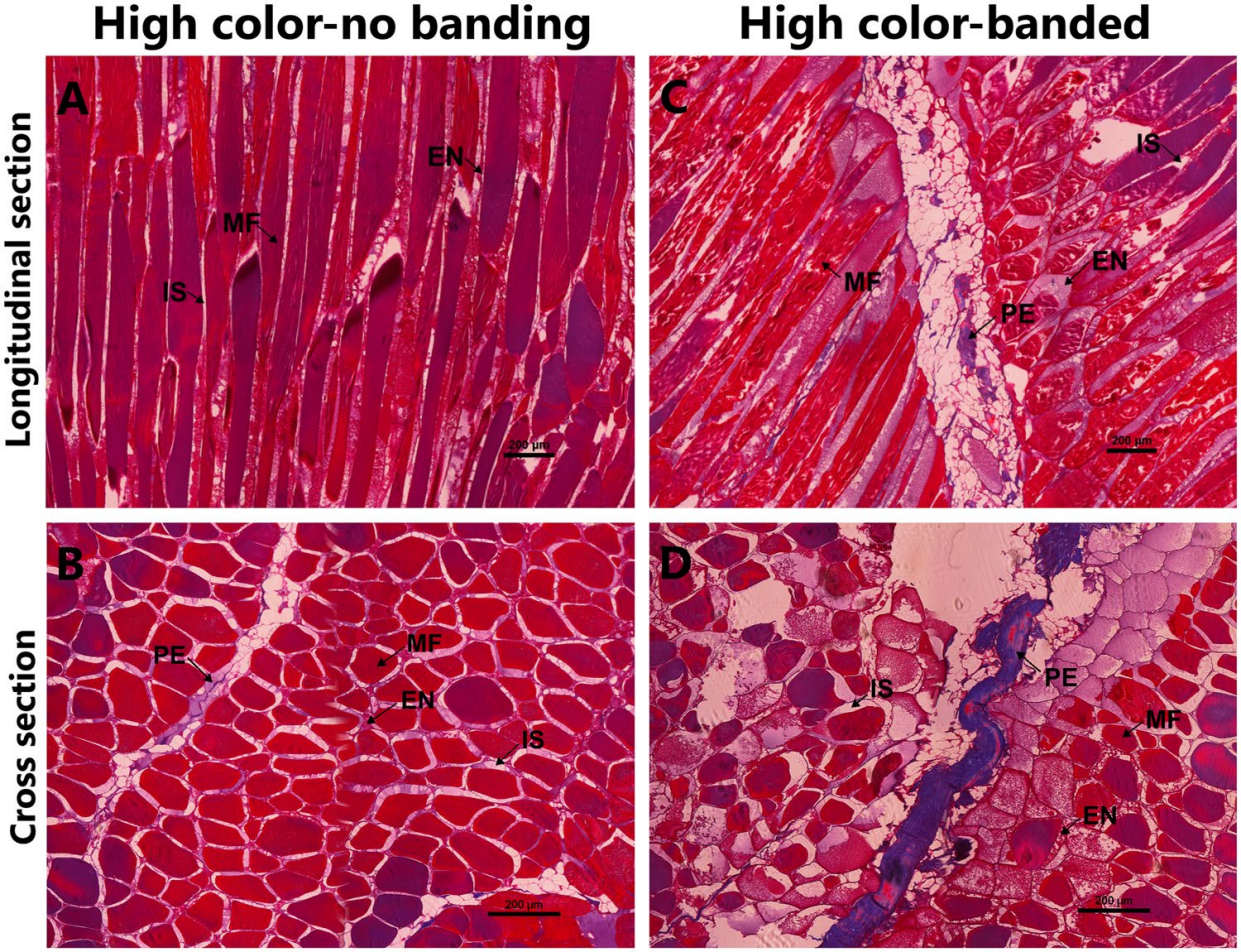
Microscopy images of the front dorsal (FD) muscle region showing structural variations among HN (**A**,**B**) and HB fish (**C**,**D**). Panels (**A**,**C**) present longitudinal sections, while panels (**B**,**D**) show cross sections, scale bar: 200 µm. *MF* muscle fiber, *PE* perimysium, *EN* endomysium, *IS* interstitial space.

Histological sections of FD and BC muscle regions were qualitatively and quantitatively analyzed. Morphological characteristics and integrity of the FD region were compared between HN and HB fish (Fig. 2), with the first displaying more intact muscle fiber (MF)-to-perimysium (PE) and MF-to-MF attachments (Fig. 2A,B) compared to the HB fish (Fig. 2C). Moreover, in HB fish muscular atrophy was observed (Fig. 2D). The average MF diameter in HB fish was significantly smaller than in HN fish (51.53 ± 1.76 µm vs 97.70 ± 1.80 µm; *P* < 0.05, Table 1). The interstitial spaces were significantly larger in HB fish when compared to HN fish (37.34 ± 1.36% vs 21.34 ± 2.21%; *P* < 0.05, Table 1). Finally, the PE was also enlarged in HB fish (Fig. 2).

**Table 1:** Quantification by light microscopy of Feret’s diameter of muscle fiber (Feret’s MFD) and percentage of interstitial space (IS) of the FD and BC region.

Index	FD region		BC region		
	HN	HB	HN	HB	*P*
MF (µm)	97.70 ± 1.80^a^	51.53 ± 1.76^b^	135.06 ± 4.13^a^	116.62 ± 4.17^a^	47.41 ± 1.00^b^
IS (%)	21.34 ± 2.21^a^	37.34 ± 1.36^b^	18.46 ± 0.39^a^	24.93 ± 0.44^ab^	40.02 ± 1.39^b^

Data are presented as mean ± SD (n = 5). Twenty measurements were recorded per sample. Data with different superscript letters in one row of each muscle region represent a significant difference between flesh color phenotypes: *HN* High color-No banding fish, *HB* High color-Banded fish, *P* Pale fish, *MF* muscle fiber, *IS* interstitial spaces. Significant differences were determined by one-way ANOVA (*P* ≤ 0.01).

**Figure 3:**
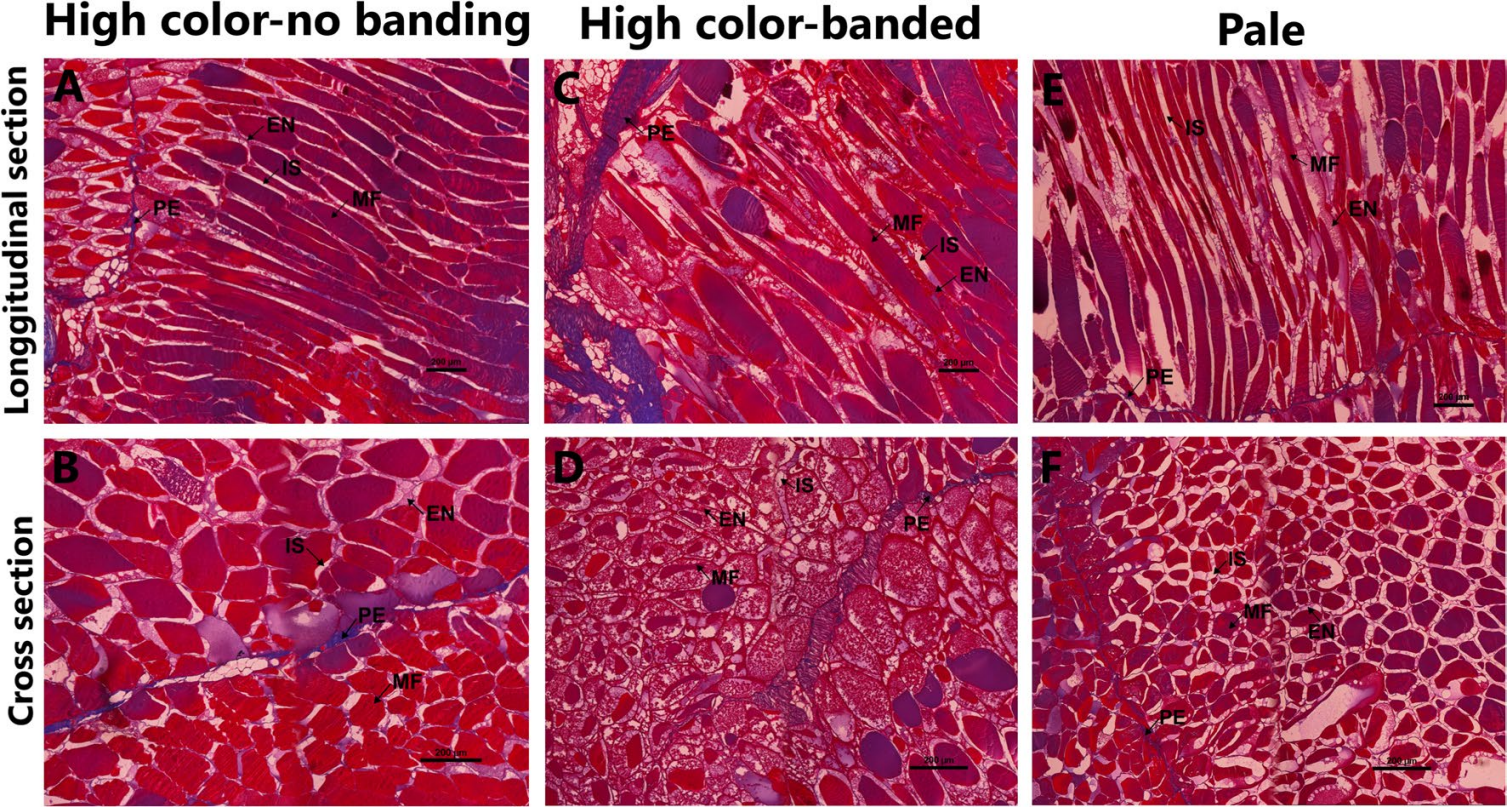
Histological sections of the back central (BC) muscle region showing structural variations among HN (**A**,**B**) HB fish (**C**,**D**) and Pale fish (**E**,**F**). Panels (**A**,**C**,**E**) present longitudinal sections, while panels (**B**,**D**,**F**) show cross sections, scale bar: 200 µm. *MF* muscle fiber, *PE* perimysium, *EN* endomysium, *IS* interstitial space.

Histological analysis of the BC muscle region showed clear structural changes between HN, HB and Pale fish (Fig. 3). In both HN and HB fish, the MF-PE attachment was tighter than in Pale fish (Fig. 3A,C,E). The interstitial spaces in HN fish were relatively small (18.46 ± 0.39 µm), while the MF diameter is comparatively larger (135.06 ± 4.13 µm, Table 1, Fig. 3B) than those in Pale fish. In HB fish, the structure appeared to be intermediary or transitional between HN and Pale fish, displaying atrophied MFs and expanded interstitial spaces (Fig. 3D). MF diameter in HB fish was similar to HN fish (*P* > 0.05) but significantly larger than Pale fish (*P* < 0.01). The interstitial spaces in HB fish were not significantly different from either HN or Pale fish (*P* > 0.05). When comparing HN and HB with Pale fish, the latter had significantly larger interstitial spaces, with most of the MF being atrophied (Fig. 3F).

#### Differentially expressed genes between three flesh color phenotypes across two muscle regions

**Figure 4:**
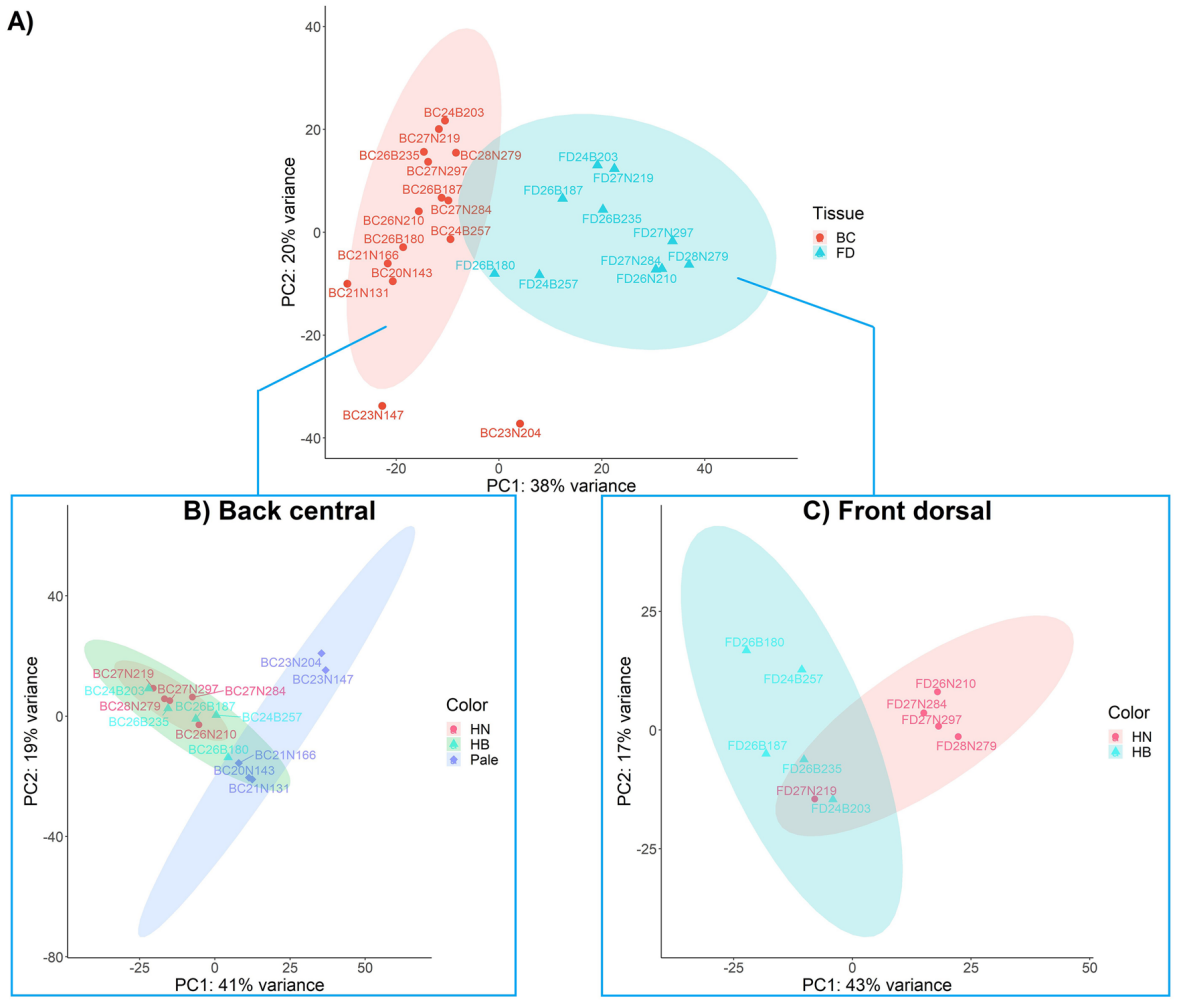
Principal component analysis (PCA) of sequencing data comparing two muscle regions and three phenotypic groups within each region. (**A**) The comparison of FD and BC regions; (**B**) Comparison of HN, HB and Pale fish in the BC region; (**C**) Comparison of HN and HB fish in the FD region. *BC* back central region, *FD* front dorsal region, *HN* high red color-no banding fish, *HB* high red color-banded fish, *Pale* fish.

The plotted PCA shows the relationships among samples and the differences between tissues and flesh color intensity groups (Fig. 4). Figure 4A shows the cluster of individual samples per tissue type, with the two clusters of FD and BC muscle regions partly overlapping, indicating variance in gene expression between the two tissues. In the BC muscle region, a complete overlap was evident between the tightly clustered HN and HB groups, which also had some overlap with the Pale group cluster (Fig. 4B). In the FD muscle region, the HN and HB clusters only partially overlap (Fig. 4C).

**Figure 5:**
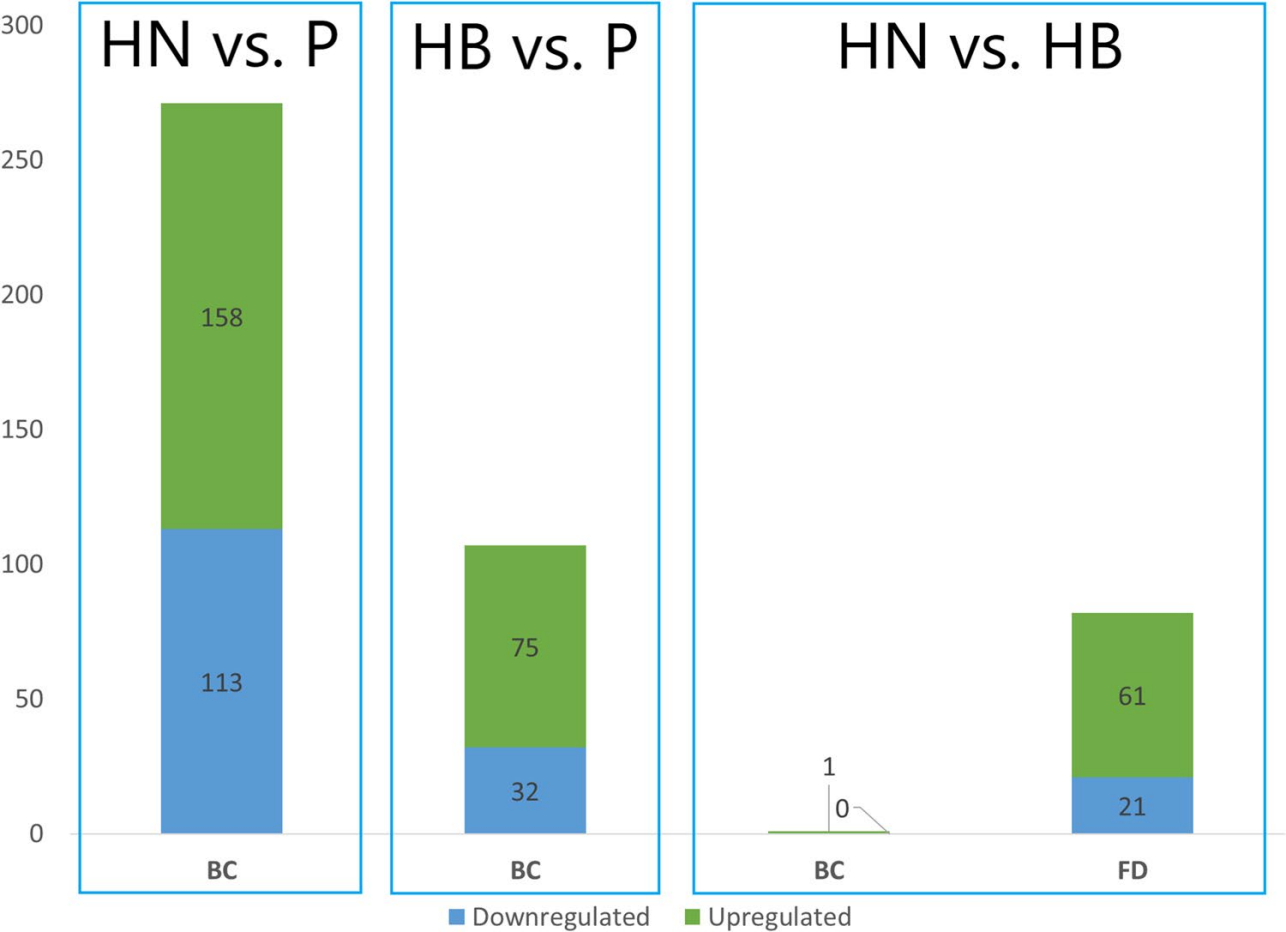
Differentially expressed genes in comparisons between the three phenotypes within each muscle region. *BC* back central region, *FD* front dorsal region, *HN* high color-no banding fish, *HB* high color-banded fish, *P* Pale fish.

**Figure 6:**
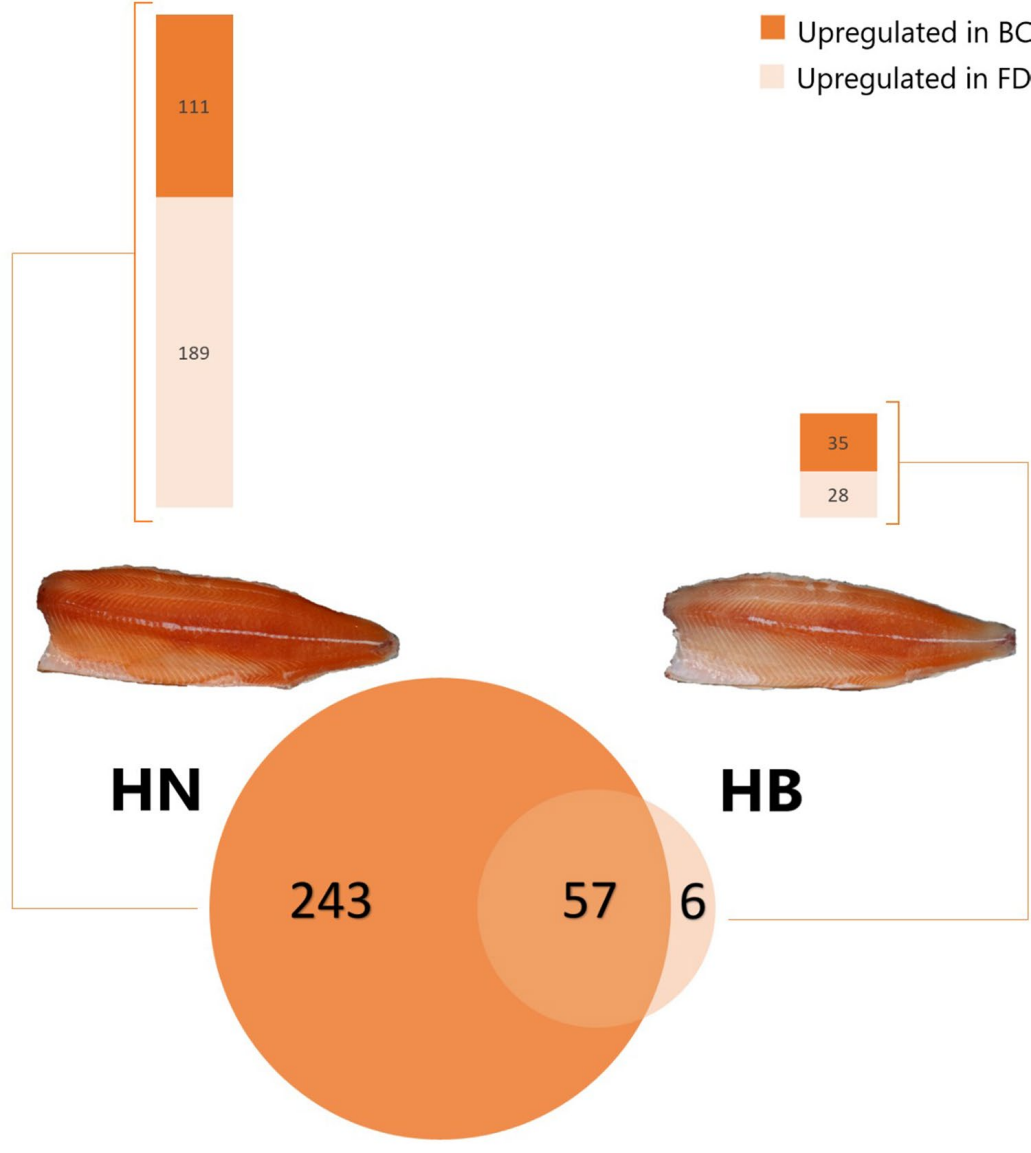
Visualization of differentially expressed genes (DEGs) between high red color-no banding (HN) and high red color-banded (HB) fish when comparing the back central (BC) and front dorsal (FD) muscle regions. Venn diagram (created by InteractiVenn^75^) showing the overlapping DEGs between HN and HB groups and the number of DEGs in HN and HB groups is presented in the bar chart.

The DEGs between the three flesh pigment phenotypes were compared within each muscle region (Fig. 5). The highest number of DEGs were found in the BC muscle region when comparing HN and Pale fish, with 271 DEGs, of which 158 and 113 were upregulated and downregulated in HN, respectively. When comparing the BC region of HB and Pale fish, the number of DEGs was 107, with 75 upregulated and 32 down regulated in HB fish. In comparing HN and HB fish, there was just one DEG detected in the BC region upregulated in HN while in the FD muscle region there were 82 DEGs, of which 61 were upregulated and 21 were down regulated in HN fish. The comparison between the two muscle regions within HN identified 300 DEGs, with 111 genes upregulated in the BC and 189 in FD. Finally, the same comparison within HB fish identified 35 genes upregulated in BC and 28 in FD, while 57 DEGs overlapped between the two red color intensity groups, HN versus HB (Fig. 6).

#### Comparative functional analysis of the three flesh color phenotypes in the front dorsal (FD) and the back central (BC) muscle regions

The KEGG pathways are presented in the Supplementary File 1. In the FD region, no KEGG pathway was found in HB fish. Two out of four pathways found in HN fish were related to amino acid metabolism. In the BC region, only fatty acid degradation, fatty acid biosynthesis and T cell receptor signaling pathways were identified in Pale fish when comparing to HN fish. In HN fish, KEGG pathways were involved in signaling, lipid, amino acid and energy metabolism. The KEGG pathways in HB fish were similar to those in HN fish, except for lipid metabolism.

Regarding the functional analysis of Gene Ontology annotation, in the FD region (where pigment depletion is more pronounced in HB fish), 24 out of 61 genes upregulated in HN fish encoded for proteins involved in muscle contraction, extracellular matrix (ECM) and calcium ion binding, while in HB fish, genes relating to lipid and fatty acid metabolism were differentially expressed (Table 2). In the BC, several highly expressed genes were related to the muscle fiber composition protein network (MFCPN), calcium ion binding, lipid metabolism, glucose metabolic and proteolytic processes, inflammation and stress responses (Table 3). Within the same color phenotype, HN or HB, gene expression profiles relating to the MFCPN and calcium-binding were distinctly different between the FD and BC muscle regions. Most myosin genes were upregulated in HN and HB fish while tropomyosin genes were upregulated in Pale fish. Actin alpha skeletal muscle 2 (*acta2*) genes were upregulated in the BC region of both HN and HB fish compared with Pale fish. Genes encoding collagens of ECM were not highly expressed among groups in the BC region. In the context of lipid metabolism, only lipase, hormone-sensitive (*lipe*) gene was upregulated in HN and HB fish compared with Pale fish, while other genes involved in lipid metabolism were upregulated in Pale fish. Genes related to glucose metabolic processes were highly expressed in Pale fish. Most genes encoding heat shock proteins and interleukin 11 (*il-11*), which is involved in inflammation, were upregulated in HN and HB fish compared to Pale fish. Gene expression of DEGs in the FD and BC regions is shown in the heatmaps (Supplementary File 2).

**Table 2:** The expression of key DEGs in the FD muscle region when comparing HN: High red color-no banding fish and HB: High red color-banded fish; downregulated genes in HN fish are highlighted in bold italics.

Gene name	Log 2-fold change
Muscle contraction and extracellular matrix	
Myosin-binding protein C, slow-type	7.53
Myosin-binding protein C, fast-type	7.24
Collagen alpha-1(I) chain* (cola1i)*	2.13
Collagen alpha-1(XI) chain* (cola1xi)*	2.40
Collagen alpha-1(XII) chain* (cola1xii)*	2.27
Collagen alpha-1(XXII) chain	2.35
Calcium ion binding	
Troponin T, fast skeletal muscle isoforms-like	2.98
Troponin I, fast skeletal muscle-like	5.55
Troponin C, skeletal muscle* (tnc)*	4.65
Parvalbumin alpha	4.63
Metabolic processes	
Leptin receptor* (lepr)*	2.32
ATP-dependent 6-phosphofructokinase, muscle type* (atpd6p)*	2.08
Lipid and fatty acid metabolism	
Apolipoprotein C-I* (apoci)*	***−2.15***
Apolipoprotein Eb* (apoeb)*	***−2.93 ***

**Table 3:** The expression of key DEGs in the BC muscle region. Downregulated genes in either HN or HB when compared to Pale are highlighted in bold italics.

Gene name	Log 2-fold change	
	HN versus *P*	HB versus *P*
Muscle contraction and extracellular matrix		
Actin, alpha skeletal muscle 2-like (*acta2*)	3.28	3.04
Myosin heavy chain, fast skeletal muscle	2.76	3.86
Myosin light chain 3, skeletal muscle	2.44	2.27
Myosin-binding protein H	2.31	2.13
Myostatin 1b (*myo1b*)	–	2.02
Thymosin beta	3.83	2.95
Tropomyosin 1 alpha	***−2.00***	–
Tropomyosin alpha-3 chain	***− 3.48***	***− 3.82***
Tropomyosin beta chain	***− 5.70***	***− 5.21***
Tropomyosin 3	***− 2.87***	***− 2.87***
Myosin-binding protein C	***− 4.64***	***− 4.25***
Troponin T	***− 4.34***	***− 4.66***
Calcium ion binding		
Parvalbumin beta 1	3.76	3.04
Parvalbumin beta-like	2.81	2.46
Parvalbumin beta 1	3.67	3.22
Lipoprotein lipase-like (*lipase*)	***− 2.76***	***− 2.39***
Fatty acid-binding protein, intestinal-like (*fabpi*)	***−2.21***	***− 2.58***
Metabolic processes		
Cathepsin L1-like (*catl*)	–	***− 3.28***
Lipase, hormone-sensitive (*lipe)*	3.21	4.33
Calsequestrin-like (*calse*)	***− 5.92***	–
Interleukin 11-like (*il-11*)	2.69	2.05
Heat shock 70 kDa protein 4 (*hsp70*)	3.92	–
Heat shock protein 30-like	3.92	–
Heat shock protein beta-1-like	2.07	–
Heat shock protein Hsp-16.1/Hsp-16.11	2.02	–
Heat shock protein 90 kDa alpha, class B member 1	***− 2.39***	–
L-serine dehydratase/L-threonine deaminase-like (*lsd/ltd*)	7.98	6.55

Comparison between the BC and FD regions in HN fish presented DEGs related to collagen, connective tissues, muscle structural integrity and calcium ion binding, while in HB fish collagens were not DEGs when comparing the two regions (Supplementary File 3). Troponin and parvalbumin gene families were downregulated in the BC region of both HN and HB fish. Perilipin 2 (*plin2*) was highly expressed only in the FD region of HN fish. The list of DEGs in the comparison between BC and FD regions in HN and HB fish is presented in Supplementary File 4.1 & 4.2).

### Validation of DEGs using qPCR

**Figure 7:**
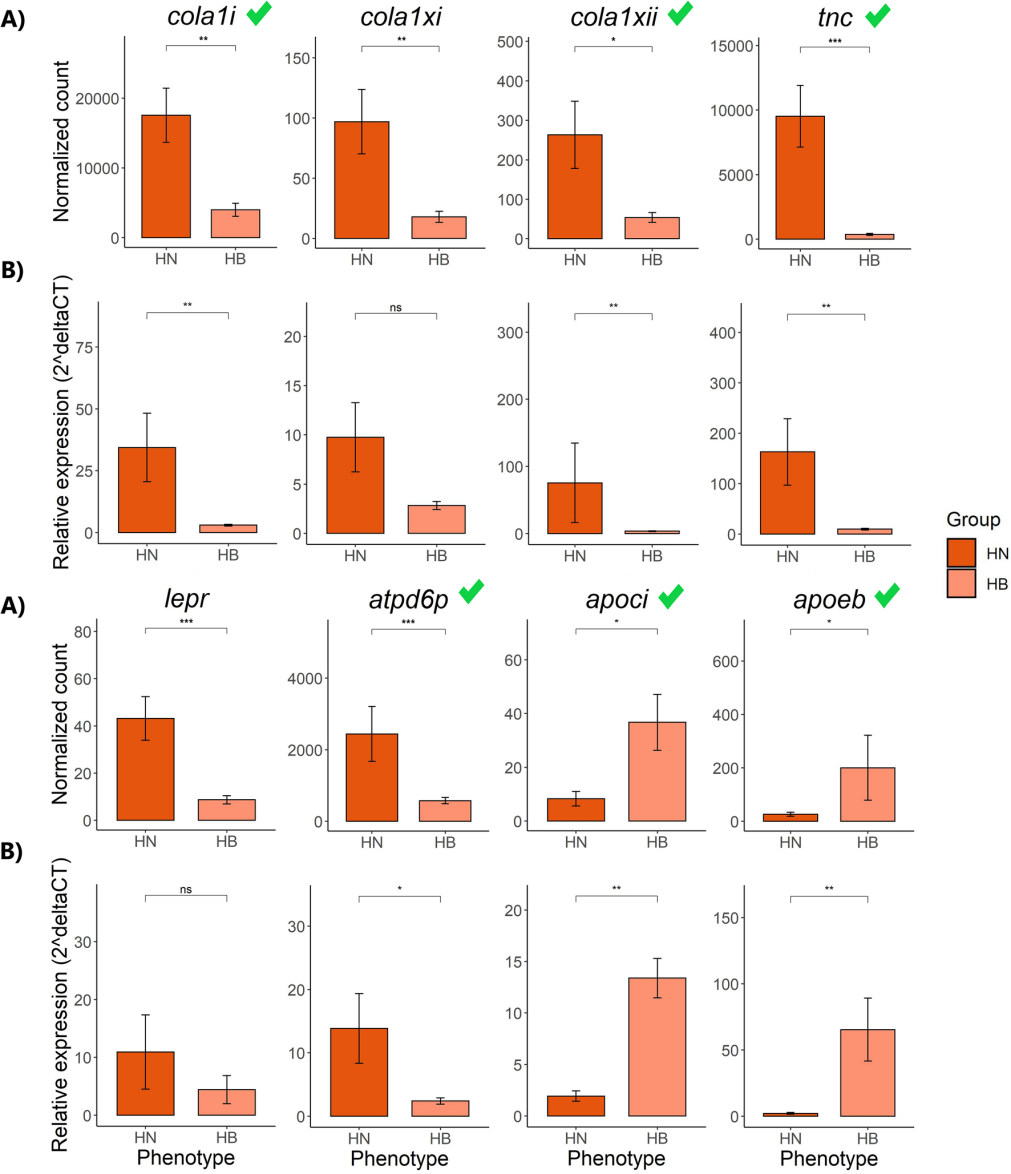
Differentially expressed genes in the FD region were validated using qPCR. Gene expression was calculated by normalized count in RNA-Seq (**A**) and relative expression in qPCR (**B**) in the comparisons of HN versus HB. An average normalized count or relative expression across transcripts is shown. Data is shown as Mean ± SD. (n = 5/sample type). Means with asterisks indicate significant differences (*P* < 0.05). A green tick indicates a confirmed DEG by qPCR.

**Figure 8:**
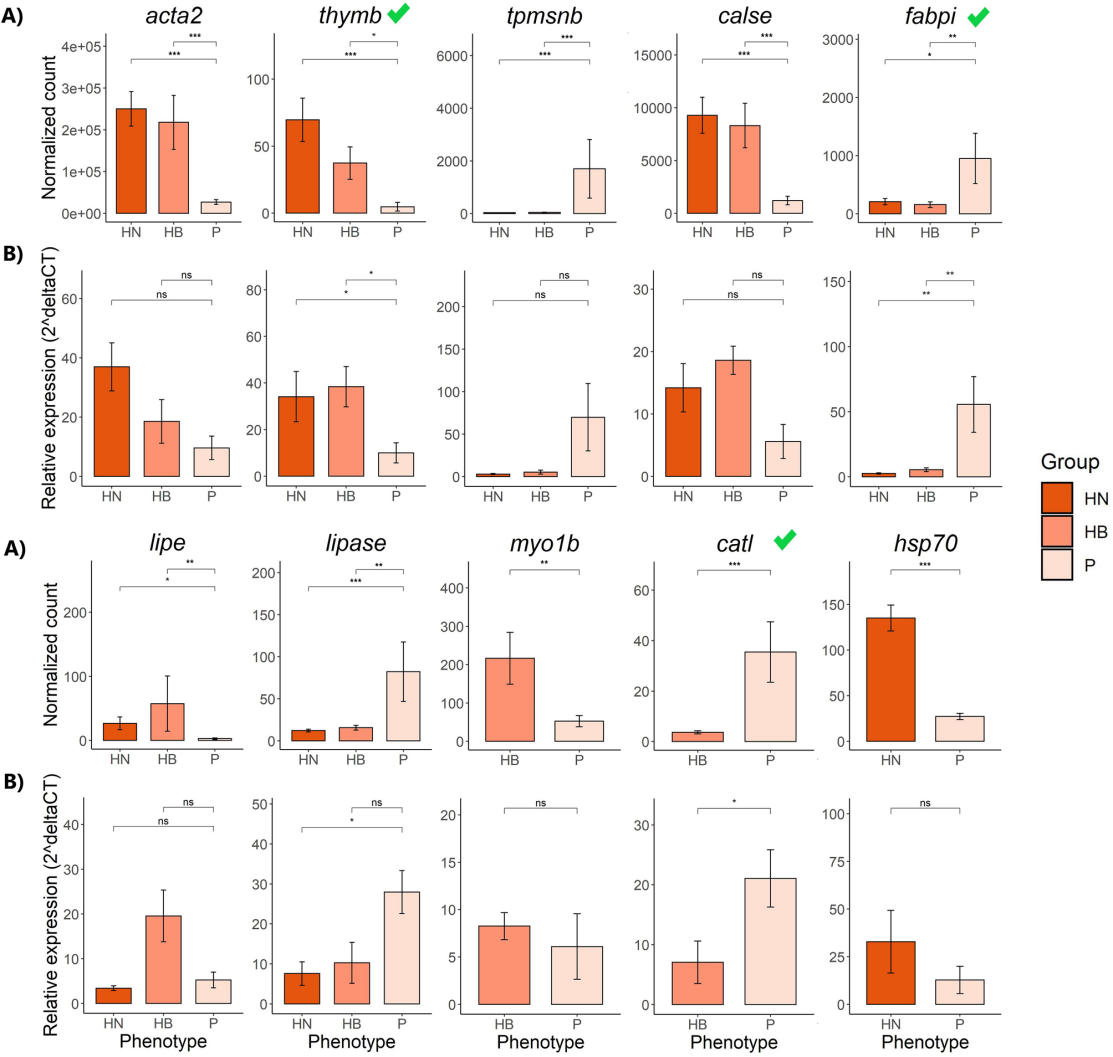
Differentially expressed genes in the back central region were validated using qPCR. Gene expression was calculated by normalized count in RNA-Seq (**A**) and relative expression in qPCR (**B**) in the comparisons of HN versus Pale and HB versus Pale. An average normalized count or relative expression across transcripts is shown. Data is shown as Mean ± SD. (n = 5/sample type). Kruskal–Wallis test was performed to verify any significant difference between the three phenotypes, then a multiple pairwise-comparison between groups was performed using pairwise Wilcoxon test. Means with asterisks indicate significant differences (*P* < 0.05). A green tick indicates a confirmed DEG by qPCR.

Eighteen DEGs were qPCR validated: *cola1i, cola1xi, cola1xii, tnc, lepr, atpd6p, apoci, apoeb* in FD, and *acta2, thymb, tpmsnb, myo1b, lipe, lipase, fabpi, catl, hsp70, calse* in BC (Supplementary File 5). The results are presented in Figs. 7 and 8 and Supplementary File 6. Overall, the trend of gene expression in both RNA-seq and qPCR was similar. The significant differential expression of 9 genes including *cola1i, cola1xii, tnc, atpd6p, apoci, apoeb, thymb, fabpi, and catl* was confirmed (*P* < 0.05). The gene expression of *cola1xi* and *lepr* in the FD region were higher in HN than the HB group (*P* > 0.05). In the BC region, the expression of *acta2, tpmsnb and lipase* was not significantly different when comparing HN and HB to Pale fish (*P* > 0.05). *thymb and calse* showed higher expression in HB than in the HN group that contrasts with RNA-seq result. *lipe, hsp70* and *myo1b* were significantly upregulated in HB fish compared with HN fish *(P* < 0.05*)*.

## Discussion

It has been ascertained that prolonged thermal stress in Atlantic salmon (*S.* *salar*), caused by high summer water temperatures, results in starvation that affects flesh pigmentation^4–7,9^. In salmon, starved for 35 up to 86 days, but at lower temperatures (approximately 11 °C and 4 °C), no effect on flesh pigmentation was observed^30,31^. In Tasmania, when water temperatures exceed 20 °C, farmed Atlantic salmon are exposed to temperatures outside their thermal tolerance range (12–18 °C)^3^. Elevated temperatures induce enhanced metabolic activity required to compensate for raised energy demand^32^, resulting in increased production of reactive oxygen species^33^ and starvation^5^. As a result of that, in Tasmania Atlantic salmon subjected to stress-induced starvation during elevated temperature periods can show either partial or complete depletion of flesh color at a variable rate between years and yearclasses^5^. Our previous genetic investigation showed that flesh colour loss has a genetic component and correlates with performance traits (e.g. size and condition factor) of fish measuread at different points during the production cycle^4^.

In our present study, following supra-optimal temperature conditions, we sampled and then categorized fish displaying three main red color intensity phenotypes, which also presented a marked difference in size. At the timepoint analyzed, HN fish were heavier than HB and Pale fish, indicating individual differences in thermal tolerance. The significant lower condition factor of Pale fish (*P* < 0.05) suggests lower feed intake compared to HN and HB fish. In response to thermal stress, reduction in HB fish feed intake and more pronounced starvation in Pale fish could explain the reduced performance.

The FD and BC muscle regions manifest the effects mentioned above in the form of differential localized discoloration (i.e. banding) or overall paleness via pigment depletion. Muscle histology of the three flesh color phenotypes conducted in this study, targeting regions of interest, clearly indicates reduced muscle integrity in HB and Pale fish (Figs. 2, 3). Transcriptomic analyses of the two muscle regions identified DEGs involved in muscle structural integrity and metabolism, which are likely responsible for pigment retention in the flesh. Genes involved in heat stress response and energy metabolism were also detected as DEGs in both regions. Here, we present the association of flesh pigmentation with muscle structural integrity, protein, lipid, fatty acid and energy metabolism and heat stress response in Atlantic salmon, which might be caused by the elevated temperature during hot summers in Tasmania.

Salmon flesh quality is defined in terms of color, texture, and flavor^27,34^. The main edible part of the fish is composed of white muscle fibers, in which myosin, the most abundant protein, is essential for the process of contractile filaments^27,35^. The number, size (diameter) and distribution of MFs, often referred to as muscular cellularity, are important determinants for flesh texture and color in fish^19,22^. A high collagen content and MF-to-MF attachments are associated with a firmer muscle structure^36,37^. Environmental conditions can impact the fish behavior and physiology and the structure and metabolic characteristics of the muscle tissue^27,38^. We have examined the muscle histology in the FD and BC regions to determine the influence of prolonged exposure to elevated water temperature on muscle structure in the three different flesh color phenotypes. Histological analysis clearly indicates that MF attachment and MF diameter are maintained in both the FD and BC regions of HN fish, while severe muscular atrophy is observed in the BC region of Pale fish (Fig. 3). In HB fish, histology displays an intermediary or transitional state between HN and Pale fish, suggesting it might represent an initiation of muscle degradation. Additionally, we found that IS is significantly expanded in both the FD and BC regions of HB fish and the BC region of Pale fish when compared to the HN fish. Previous studies determined that MF-to-MF and PE-to-MF attachments are associated with muscle quality^36,39,40^ and that MF detachment is linked with reduced flesh firmness in Atlantic salmon^40^. Therefore, although not measured in the current study, it is likely that flesh texture in HB and Pale fish is softer compared to HN fish, which deems further investigation.

In salmon, astaxanthin was demonstrated to bind to muscle proteins^41^ and a positive relationship between flesh pigmentation and fiber density was proposed^22^. In a recent study, Grünenwald, et al.^7^ proposed that localized carotenoid retention can be attributed to the variety of muscle fiber proteins in the Atlantic salmon fillet. Therefore, the amount of astaxanthin deposited and bound to MF proteins might decrease due to reduced quantity of muscle MF proteins and structural degradation. This suggests that pigment mobilization might occur not only as a result of their metabolic utilization to countermeasure thermal stress but also due to forced release following tissue degradation and binding sites disruption.

The MFCPN is among the essential components that impact muscle structural coherence in salmonids and other teleosts^21,22,27,42,43^. A more pronounced effect of genes encoding MFCPN factors was recorded in both tested muscle regions; genes transcribing muscle fiber proteins and intracellular calcium ion (Ca^2+^)-binding proteins were highly expressed in the FD region of HN fish compared to HB fish (17 versus 3 genes) and in the BC region of both HN (23 versus 9 genes) and HB fish (24 versus 10 genes), when compared with Pale fish. This striking positive correlation between flesh pigmentation and MFCPN is in agreement with previous findings^7,22,41,43^, signifying that flesh color correlates with MFCPN availability to bind the pigments. When comparing HN and HB fish to Pale fish, genes encoding myosin muscular proteins and actin muscle proteins (actin-alpha skeletal muscle-2, *acta2*) and *il-11* gene, which is involved in inflammation, were significantly downregulated in the BC region of Pale fish. In addition, cathepsin L (*catl*), which is involved in proteolysis, was upregulated in the BC of Pale fish (Fig. 8), while heat shock proteins, particularly *hsp70,* were highly expressed in HN fish. Actomyosin, the major contractile protein complex in the white muscle of salmonids, is composed of F-actin combined with myosin, which loosely binds to astaxanthin^15–17,44^. In Atlantic salmon, carotenoids were detected in protein fractions containing F-actin, including actomyosin, actin and F-actin fractions, but not found in myosin protein fractions^18^. In actomyosin, myosin II is composed of myosin heavy chains and light chains that are responsible for muscular cell contraction^45^ and are mostly involved in thermal acclimation, leading to the altered expression of myosin heavy and light chains^46^. In mice, it was demonstrated that *il-11* expression inhibits inflammation and stimulates airway fibrosis with the intensified accumulation of interstitial collagens and myocytes and myofibroblasts^47,48^. Moreover, reduction in heat shock proteins and increase of cathepsin activities were correlated with dissociation of myosin and actin in the actomyosin complex in grass carp^49^. Disassembly of muscle fibers and elevation of cathepsin activities reduced the firmness of rainbow trout flesh^42^. These findings are consistent with our current transcriptomic results, pointing to the disassembly of myosin and actin in the actomyosin complex and proteolysis of myosin and actin proteins or actomyosin in Pale fish exposed to thermal stress. The proteolytic process might be compensatory for an increase in energy demand in the response to thermal stress in Pale fish. When looking at our histology results, in Pale fish proteolysis might be responsible for loss of flesh firmness (although not measured) as well as for the deterioration of actin, possibly leading to the loss of carotenoids bound to F-actin muscle fiber.

In HB fish, both histological and transcriptomic analyses indicate that the changes in muscle integrity mainly occurred in the FD region, in keeping with its involvement in the localized pigment depletion, which is clearly visually observed in that phenotype (i.e. banding). Particularly, genes encoding myosin proteins were downregulated in the FD region compared to HN fish. In line with that discussed above concerning Pale fish, the downregulation of myosin in the FD region of HB fish could affect the actomyosin complex, leading to weakening in the binding of actin or actomyosin and carotenoids with consequent loss of them. Moreover, both the deterioration of ECM in the FD region in HB fish, when compared to HN fish (Fig. 2A,C) and the downregulation of ECM collagen genes (*cola1i, cola1xi and cola1xii*, Fig. 7) were identified in the FD region of HB fish but not in the BC region. Collagen is the most abundant protein in connective tissues of fish muscle and plays a critical role in supporting muscle integrity^50,51^. These studies indicate that in the condition of low collagen abundance, binding of MF-PE could be disrupted, leading to decreased muscle structural integrity, resulting in loose binding of carotenoid-binding proteins and carotenoids. In light of these studies, our findings suggest that the FD region can experience loss of carotenoid pigments to a greater extent than the BC region due to the potential impact of reduced collagen abundance.

**Figure 9:**
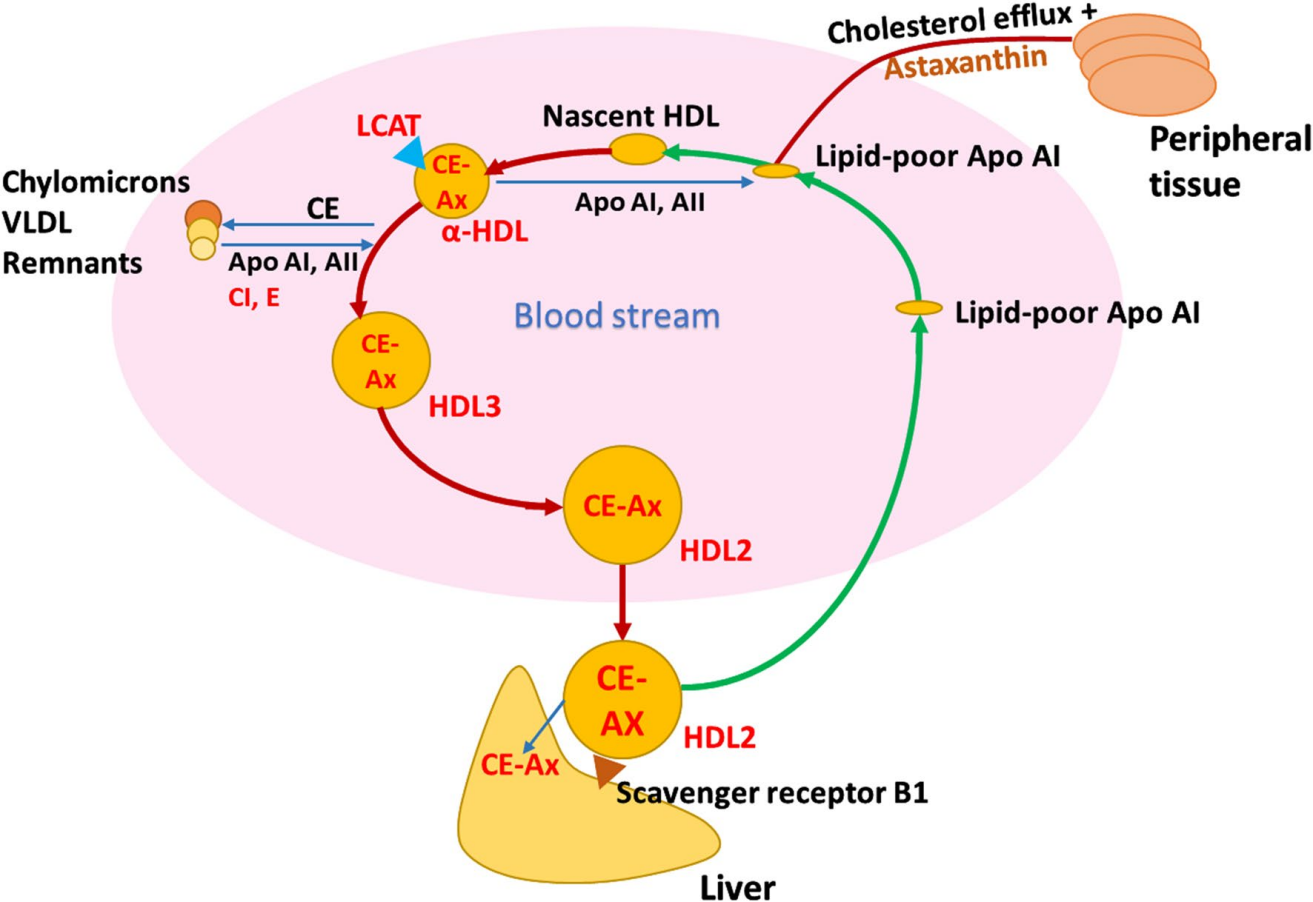
Proposed model for astaxanthin transport, accompanied by cholesterol in Reverse cholesterol transport from peripheral tissues to the liver by high-density lipoprotein (HDL). HDL particles carry cholesterol from peripheral tissues to the liver and also embed cholesteryl esters (CEs) by interchanging them for triglycerides (TG) with TG-rich lipoproteins. Gathering of CEs transforms the particle to spherical HDL3. HDL3 transfers CE, ApoA, ApoC and ApoE to enlarge its particle size, which matures into HDL2. HDL2 then disposes of CE by docking into the scavenger receptor type B class I (BI). In this way, HDL enables the maintenance of extracellular cholesterol pool. (Reverse cholesterol transport by high-density lipoprotein (HDL) modified from^55^). The potential impacted pathways inducing the loss of astaxanthin from muscle were highlighted in red color. *HDL* high-density lipoprotein, *VLDL* very low-density lipoprotein, *CE-Ax* cholesteryl ester-Astaxanthin, *Apo AI* apolipoprotein AI, *AII* apolipoprotein AII, *CI* apolipoprotein CI, *E* apolipoprotein E, *LCAT* lecithin:cholesterolacyl transferase.

In the context of lipid and fatty acid metabolism, the leptin receptor *(lepr)* gene was downregulated, while apolipoprotein genes (C-I, *apoci* and Eb, *apoeb*) were upregulated in the FD region of HB fish compared to HN fish (Fig. 7). *lepr* upregulation associates with enhancement of growth and lipid deposition in Atlantic salmon^52^. This may indicate that in HB fish, growth performance and fat deposition in the flesh were reduced, possibly caused by the thermal stress. Apolipoproteins E and C-I (ApoE and ApoCI) are proteins that play a role in the transport and metabolism of cholesterol and other lipids^53^  (Fig. 9). ApoE is found on the surface of low-density lipoprotein (LDL) particles which are involved in the transport of cholesterol and other lipids from the liver to other parts of the body. ApoCI is the main structural component of high-density lipoprotein (HDL) and very-low-density lipoprotein (VLDL)^54^, which are involved in the reverse transport of cholesterol and other lipids from the periphery to the liver^53,55^. ApoCI also plays a role in regulating the activity of enzymes involved in lipid metabolism, such as hepatic lipase (HL) and an activator of lecithin:cholesterolacyl transferase (LCAT)^54^. In addition, apoE and ApoCI have other functions, such as participating in the repair of tissue injury, immunoregulation, and modulating the immune response and regulating cell growth and differentiation^56,57^. Finally, it has been suggested that the upregulation of ApoE and ApoCI in certain fish may enhance the synthesis of cholesterol esters in HDL particles^56,57^ which could lead to the depletion of pigment in the flesh of fish due to the mobilization of astaxanthin. We suggest that in HB fish the loss of pigmentation in the FD area (i.e. banding) is also linked to lipid metabolism and transport which are enhanced to respond to thermal stress.

In the BC region, the upregulation of the genes encoding lipoprotein lipase (*lipase*) and fatty acid-binding protein (*fabpi*) was identified only in Pale fish (Fig. 8). *lipase*, which encodes a triacylglycerol lipase, catalyzes the hydrolysis of triglycerides free fatty acids chylomicrons^58^. Furthermore, the proteins encoded by fatty acid-binding protein gene family play a role as transporters of fatty acids through the cytoplasm to the peripheral organs and liver in Atlantic salmon and other fish species^59^. In the proposed carotenoid dynamic of salmonids, carotenoids can be mobilized accompanied by lipid molilization^11,58,60,61^. Also, increased carotenoids absorption associates with some dietary lipid elements, such as fatty acids, lipid and cholesterol level^62–65^. Hence, due to actin degradation, carotenoids may be freed and bind to fatty acids in the muscle. Because the flesh pigment depletion did not link to oxidative stress in starved Atlantic salmon exposed to prolonged hot summer^6,7^, we hypothesize that free astaxanthin could be mobilized and lost through the metabolic pathway of lipid in the BC muscle. Our current results suggest intensified carrying and diffusion of fatty acids accompanied by carotenoids in the Pale fish. Hence, during the starvation period in summer, Pale fish might have increased the intensity of proteolysis and of lipid and fatty acid metabolism to provide energy for maintenance of physiological activities. Therefore, depletion of flesh pigmentation occurs via concurrent catabolism of fatty acids and astaxanthin.

In our hypothesis, the pigment depletion and mobilization via fatty acid metabolism initially begins in the FD, an area of the fillet known to have higher lipid content^66^, followed by the BC region. Proteolysis can occur in parallel or later, leading to further discoloration through actual deterioration of binding sites and loss of pigments. Likely, the pigment depletion happens in the BC when the FD region is exhausted, or maybe it is just delayed or happens simultaneously to a minor extent. As a result, flesh discoloration occurs in the FD region of the HB fish and in both regions in the Pale fish.

## Conclusions

Thermal stress resulting from prolonged elevated water temperatures considerably impacted Atlantic salmon in our present study. As a consequence, starvation is likely to have happened for an extended time period. Starvation negatively influence (at individually different extent levels) energy metabolism, resulting in either localized discoloration from the front dorsal muscle region (i.e., banding) or in entire discoloration of the whole fillet (paleness). Furthermore, fish subjected to starvation probably have enhanced catabolic process of protein and lipid, compensating for energy loss due to nutrient and energy deficiency.

In the muscle tissues, genes differentially expressed were primarily associated with carotenoids-binding proteins, the MFCPN, and the collagen family in the ECM. At the gene level, flesh pigment reduction is connected to muscle structural integrity by degradation of myosin proteins and actin alpha skeletal muscle-2 in actomyosin complex and protein and lipid metabolism. A degradation of carotenoid-binding proteins can be caused by the proteolytic process in the muscle, involving cathepsin L, setting free carotenoids then transported together with lipoproteins and fatty acids as part of the lipid metabolism. We suggest that *acta2* plays an important function in binding carotenoids and flesh color formation in Atlantic salmon. Our findings prove that thermal stress is accountable not only for flesh pigment depletion but also that the process is likely to cause loss of muscle integrity, as suggested by our muscle histology studies. From our results, a link between lipid content and carotenoid retention in salmon flesh is hypothesized. A further study on muscle structural integrity combined with biochemistry analysis in Atlantic salmon exposed to prolonged elevated water temperatures should be carried out to expand on our current findings.

## Materials and methods

### Fish sampling

Experimental fish were all female diploids from the same smolt-cohort and cultured in Tasmania since May 2018, as described in our previous study^10^. Supplement of a carotenoid mixture including astaxanthin and canthaxanthin was adjusted from 75 ppm (astaxanthin/canthaxanthin: 50/25) through the winter to 80 ppm (40/40) during the summer until harvest. In the summer of 2019, the water temperature (measured at 5 m depth) ranged between 18 °C and 21 °C^4^. After the summer, the sampling was carried out on the 2nd and 3rd of April, 2019 when the water temperature was around 10 °C, from 100 fish kept under similar conditions in two sea cages. Following the classification of the flesh color phenotypes, as defined in our previous study^10^ and Supplementary File 7, three flesh phenotypes were observed: high red color-no banding (HN, n = 39), high red color-banded (HB, n = 25) and Pale color (Pale, n = 13). From the classified groups above, fifteen representative fish were chosen for transcriptomic analysis from the three groups (n = 5 per group). Statistical analysis was conducted on the average weight and condition factor of the three phenotypes. One gram of muscle tissue from the front dorsal (FD) and the back central (BC) regions of the fillet (Fig. 1) were collected from the fifteen fish. Due to the similar flesh color of HB and Pale fish in the FD region, and minimal difference between the two muscle regions (BC and FD) in Pale fish (Fig. 1), FD samples of the Pale fish were excluded from the transcriptomic analysis. Samples were snap-frozen into liquid nitrogen and then stored at − 80 °C until analysis. From the selected fish, n = 25 muscle samples (10 samples of FD from HN and HB groups and 15 samples of BC from HN, HB and Pale groups) were fixed in Bouin’s solution (Cat/No. HT10132, Sigma-Aldrich, USA) for histological analysis. All experimental protocols were approved and performed in accordance with the University of the Sunshine Coast (USC) animal ethics approval (AN/E/16/12). We confirm the study was carried out in compliance with the ARRIVE guidelines.

### Quantitative histological analysis of two muscle regions across flesh color phenotypes

Fixed muscle samples were sent to QIMR Berghofer Medical Research Institute (Queensland, Australia) for histological processing. Microscopic images of Masson’s trichrome-stained longitudinal and transverse sections were captured using Nikon Eclipse Ti microscope and Nikon NIS-Elements AR software. Following that, 20 random muscle fibers from each sample were measured to achieve a minimal Feret’s muscle fiber diameter^67^. Percentage of interstitial spaces were quantified using light microscopy by counting 20 attachments for each sample using Fiji, an image processing package of ImageJ2^68^.

### Transcriptomic analysis

RNA was extracted from the two muscle regions using TRIzol™ Reagent (Invitrogen™, Cat.No 15596026, Life Technologies Corporation, CA, USA). The quantity and integrity of the extracted RNA were determined using NanoDrop 2000 spectrophotometer (Thermo Fisher Scientific, DE, USA) and gel electrophoresis. A total of 25 qualified and quantified RNA samples were sent to Novegene (Hongkong) for TruSeq library preparation and sequencing with Illumina HiSeq2500. The sequencing datasets were submitted to the NCBI SRA database in the BioProject Accession number PRJNA706530, ID 706530—BioProject—NCBI (nih.gov).

Approximately 21 million 150-bp paired-end raw reads were acquired from each library. All raw reads were quality assessed using FastQC v.0.11.6 (http://www.bioinformatics.babraham.ac.uk/projects/fastqc/) and Trimmomatic^69^ to examine and trim low quality reads with the parameters: ILLUMINACLIP:TruSeq3-PE-2.fa:2:30:10 HEADCROP:8 LEADING:5 TRAILING:5. Cleaned reads were aligned to the *S.* *salar* genome (Atlantic Salmon Genome—ICSASG_v2, https://www.ncbi.nlm.nih.gov/assembly/GCF_000233375.1/#/def) using HISAT2 v.2.1.0 with default parameters^70^. HTSeq v.0.9.1^71^ was used to count reads applying default parameters. For each comparison, only genes with a minimum of 10 mapped reads were considered.

### qPCR validation

The fifteen selected fish used in transcriptome analysis were chosen to validate the digitally-calculated gene expression results. qPCR was performed as previously described^72^. Briefly, ProbeFinder Assay Design web-based software was used to design primers (https://lifescience.roche.com/en_au/brands/universal-probe-library.html#assay-design-center). cDNA templates were prepared using Bioline cDNA Synthesis kit (Cat. No. BIO-65043) and mixed with primers, FastStart Universal Probe Master (Rox; Cat/No. 04914058001, Roche Diagnostics GmbH, MA, Germany) and Universal ProbeLibrary Probe (Roche Diagnostics GmbH, MA, Germany). Relative quantification was done by equilibrating to the level of 18S rRNA, the housekeeping gene per sample and the sample with the lowest value (2^−∆∆CT^). The stability of 18S gene was checked as presented in Supplementary File 8.

### Statistics

To identify differences in average weight and condition factor and then from the image analysis of muscle histological samples, in muscle fiber diameter and percentage of interstitial spaces a Kruskal–Wallis non-parametric test of multiple comparisons between flesh color phenotypes was performed using SPSS version 26.0 (IBM SPSS Statistics for Windows, Armonk, NY, USA).

For the transcriptomic analysis, a principal component analysis (PCA) was used to plot and visualize the distribution of data between tissues and red color intensity groups within each tissue. Differentially expressed genes (DEGs) were analyzed to compare the three phenotypes using DESeq2 in R (version 3.5.3)^73^. A gene was considered a DEG between two conditions when the following applied: |log2(fold change)|≥ 2, *p*-adjusted value (false discovery rate, FDR) ≤ 0.05. In the muscle samples, the FD and BC regions within each group (HN and HB) were compared. Gene annotation was searched from *S. salar* genome annotation file (gff. file). In addition, Gene Ontology (GO) annotation was retrieved by Functional Analysis after a Blast search in Blast2GO 5.2^74^. Following that, the enzyme code of KEGG pathway was annotated. An KEGG pathway which involved at least two upregulated or downregulated genes was considered as valid.

For qPCR validation, statistical analysis of the resulting relative expression of chosen DEGs was examined to compare means of multiple groups, followed by Wilcoxon (for two non-parametric groups) or Kruskal–Wallis test (for multiple non-parametric groups), with *P* < 0.05 considered as statistically significant.

### Ethics declarations

All experimental protocols were approved and performed in accordance with the University of the Sunshine Coast (USC) animal ethics approval (AN/E/16/12).

## Supplementary Information

Supplementary Information 1.

Supplementary Information 2.

Supplementary Information 3.

Supplementary Information 4.

Supplementary Information 5.

Supplementary Information 6.

Supplementary Information 7.

Supplementary Information 8.

## Data Availability

The datasets supporting the conclusions of this article are available in the BioProject Accession number PRJNA706530, ID 706530—BioProject—NCBI (nih.gov).
