# Supplementary material for: Histological and transcriptomic analysis of muscular atrophy associated with depleted flesh pigmentation in Atlantic salmon (Salmo salar) exposed to elevated seawater temperatures
Source: Sci Rep. 2023 Mar 14;13:4218. doi: 10.1038/s41598-023-31242-2 (PMC10015013; doi:10.1038/s41598-023-31242-2)
Supplement: Supplementary file 1 — Supplementary Information 1. [file 41598_2023_31242_MOESM1_ESM.pdf]

Histological and transcriptomic analysis of muscular atrophy associated with depleted flesh pigmentation in Atlantic salmon (*Salmo salar*) exposed to elevated seawater temperatures

Thu Thi Minh Vo[a,b,d,*] thu.vo@research.usc.edu.au, Gianluca Amoroso[c] gianluca.amoroso@utas.edu.au, Tomer Ventura[a,b,*] tventura@usc.edu.au, and Abigail Elizur[a,*]aelizur@usc.edu.au

[a] Centre for Bioinnovation, [b] School of Science, Technology and Engineering, University of the Sunshine Coast, 4 Locked Bag, Maroochydore DC, Queensland 4558, Australia

[c] Institute for Marine and Antarctic Studies, University of Tasmania, Private Bag 49, Hobart, Tasmania 7001, Australia

[d] School of Biotechnology, International University, Vietnam National University, 700000 Ho Chi Minh City, Vietnam

***** Corresponding authors: Prof Abigail Elizur (aelizur@usc.edu.au) and A/Prof Tomer Ventura (tventura@usc.edu.au), Thu Thi Minh Vo (thu.vo@research.usc.edu.au)

Supplementary File 1. The list of KEGG pathways was annotated in multiple comparisons across the front dorsal and back central muscle regions

Comparison	Pathway	Enzyme	DEGs involved
**Front dorsal: HN versus HB fish**			
**HN fish**	mTOR signaling pathway	ec:2.7.11.24 - protein kinase	SRSF protein kinase 2-like
		ec:2.7.11.11 - protein kinase	cGMP-dependent protein kinase 1-like
	Galactose metabolism	ec:2.7.1.11 - phosphohexokinase	ATP-dependent 6-phosphofructokinase, muscle type-like
	Alanine, aspartate and glutamate metabolism	ec:3.4.17.21 - carboxypeptidase II	ATP/GTP binding protein 1<br>inactive carboxypeptidase-like protein X2
	Pyrimidine metabolism	ec:3.1.3.5 - uridine 5'-nucleotidase	troponin T, fast skeletal muscle isoforms-like<br>5'-nucleotidase domain-containing protein 1-like
**HB fish**	N.D	N.D	N.D
**Back central**<br>**HN versus Pale fish**			
**HN fish**	Pantothenate and CoA biosynthesis	ec:4.1.1.11 - 1-decarboxylase	glutamate decarboxylase-like 1<br>acidic amino acid decarboxylase GADL1-like

Glycosaminoglycan biosynthesis - chondroitin sulfate / dermatan sulfate	ec:2.8.2.33 - 4-sulfate 6-O-sulfotransferase<br>ec:2.8.2.5 - 4-sulfotransferase	carbohydrate sulfotransferase 11carbohydrate sulfotransferase 15-like
Methane metabolism	ec:3.1.3.3 – phosphatase<br>ec:4.2.1.11 - hydratase	Phosphoserine phosphatase<br>enolase 3-1
Sphingolipid metabolism	ec:2.3.1.24 - N-acyltransferase<br>ec:3.1.3.4 – phosphatase<br>ec:3.1.6.1 - sulfatase	ceramide synthase 1-like<br>lipid phosphate phosphatase-related protein type 5-like<br>arylsulfatase D-like
Taurine and hypotaurine metabolism	ec:4.1.1.29 – decarboxylase<br>ec:4.1.1.15 - decarboxylase	glutamate decarboxylase-like 1<br>acidic amino acid decarboxylase GADL1-like
Inositol phosphate metabolism	ec:3.1.3.56 - 5-phosphatase<br>ec:3.1.3.36 - 5-phosphatase<br>ec:2.7.1.68 - 5-kinase,<br>ec:5.3.1.1 - isomerase	inositol polyphosphate 5-phosphatase K-like<br>phosphatidylinositol 4-phosphate 5-kinase-like protein 1<br>triosephosphate isomerase 1b
beta-Alanine metabolism	ec:4.1.1.11 - 1-decarboxylase<br>ec:6.3.2.11 – synthase	glutamate decarboxylase-like 1<br>acidic amino acid decarboxylase GADL1-like

		ec:4.1.1.15 - decarboxylase	
	Glycine, serine and threonine metabolism	ec:3.1.3.3 – phosphatase<br>ec:4.3.1.17 - ammonia-lyase<br>ec:2.1.4.1 – amidinotransferase<br>ec:1.1.1.103 - 3-dehydrogenase<br>ec:4.3.1.19 - ammonia-lyase	Phosphoserine phosphatase<br>L-serine dehydratase/L-threonine deaminase-like<br>glycine amidinotransferase, mitochondrial-like<br>L-threonine 3-dehydrogenase, mitochondrial-like
	Alanine, aspartate and glutamate metabolism	ec:6.3.5.4 - synthase (glutamine-hydrolysing)<br>ec:4.1.1.15 - decarboxylase	asparagine synthetase [glutamine-hydrolyzing]-like<br>glutamate decarboxylase-like 1
	Fructose and mannose metabolism	ec:3.1.3.46 - 2-phosphatase<br>ec:5.3.1.1 - isomerase	probable fructose-2,6-bisphosphatase TIGAR A<br>triosephosphate isomerase 1b
	Arginine and proline metabolism	ec:2.6.1.13 – aminotransferase<br>ec:2.1.4.1 – amidinotransferase<br>ec:6.3.2.11 - synthase	ornithine aminotransferase, mitochondrial-like<br>glycine amidinotransferase, mitochondrial-like<br>carnosine synthase 1-like

**Pale fish**	Fatty acid degradation	ec:6.2.1.3 - ligase	long-chain-fatty-acid--CoA ligase ACSBG2-like
	T cell receptor signaling pathway	ec:2.7.10.2 - protein-tyrosine kinase, ec:3.1.3.16 - phosphatase	focal adhesion kinase 1-like<br>Dysferlin-interacting protein 1
	Fatty acid biosynthesis	ec:6.2.1.3 - ligase	long-chain-fatty-acid--CoA ligase ACSBG2-like
**HB fish versus Pale fish**			
**HB fish**	Butanoate metabolism	ec:4.1.1.15 - decarboxylase	glutamate decarboxylase-like 1<br>acidic amino acid decarboxylase GADL1-like
	Taurine and hypotaurine metabolism	ec:4.1.1.15 – decarboxylase<br>ec:4.1.1.29 - decarboxylase	glutamate decarboxylase-like 1<br>acidic amino acid decarboxylase GADL1-like
	Pantothenate and CoA biosynthesis	ec:4.1.1.11 - 1-decarboxylase	glutamate decarboxylase-like 1<br>acidic amino acid decarboxylase GADL1-like
	beta-Alanine metabolism	ec:4.1.1.15 – decarboxylase<br>ec:4.1.1.11 - 1-decarboxylase	glutamate decarboxylase-like 1<br>acidic amino acid decarboxylase GADL1-like

	PD-L1 expression and PD-1 checkpoint pathway in cancer	ec:3.1.3.16 - phosphatase	protein phosphatase 1 regulatory subunit 3G-like<br>myosin heavy chain, fast skeletal muscle-like
	Glycine, serine and threonine metabolism	ec:4.3.1.17 - ammonia-lyase<br>ec:1.1.1.103 - 3-dehydrogenase<br>ec:2.1.4.1 – amidinotransferase<br>ec:4.3.1.19 - ammonia-lyase	L-serine dehydratase/L-threonine deaminase-like<br>L-threonine 3-dehydrogenase, mitochondrial-like<br>glycine amidinotransferase, mitochondrial-like
	Valine, leucine and isoleucine biosynthesis	ec:4.3.1.19 - ammonia-lyase	L-serine dehydratase/L-threonine deaminase-like
	Alanine, aspartate and glutamate metabolism	ec:6.3.5.4 - synthase (glutamine-hydrolysing)<br>ec:4.1.1.15 - decarboxylase	asparagine synthetase [glutamine-hydrolyzing]-like<br>glutamate decarboxylase-like 1<br>acidic amino acid decarboxylase GADL1-like
	Th1 and Th2 cell differentiation	ec:3.1.3.16 - phosphatase	protein phosphatase 1 regulatory subunit 3G-like<br>myosin heavy chain, fast skeletal muscle-like

	T cell receptor signaling pathway	ec:3.1.3.16 - phosphatase	protein phosphatase 1 regulatory subunit 3G-like<br>myosin heavy chain, fast skeletal muscle-like
	Arginine and proline metabolism	ec:2.6.1.13 – aminotransferase<br>ec:2.1.4.1 – amidinotransferase<br>ec:3.4.11.5 - aminopeptidase	ornithine aminotransferase, mitochondrial-like<br>glycine amidinotransferase, mitochondrial-like
**Pale fish**	N.D	N.D	N.D
