# Supplementary material for: Histological and transcriptomic analysis of muscular atrophy associated with depleted flesh pigmentation in Atlantic salmon (Salmo salar) exposed to elevated seawater temperatures
Source: Sci Rep. 2023 Mar 14;13:4218. doi: 10.1038/s41598-023-31242-2 (PMC10015013; doi:10.1038/s41598-023-31242-2)
Supplement: Supplementary file 3 — Supplementary Information 3. [file 41598_2023_31242_MOESM3_ESM.pdf]

Histological and transcriptomic analysis of muscular atrophy associated with depleted flesh pigmentation in Atlantic salmon (*Salmo salar*) exposed to elevated seawater temperatures

Thu Thi Minh Vo[a,b,d,*] thu.vo@research.usc.edu.au, Gianluca Amoroso[c] gianluca.amoroso@utas.edu.au, Tomer Ventura[a,b,*] tventura@usc.edu.au, and Abigail Elizur[a,*]aelizur@usc.edu.au

[a] Centre for Bioinnovation, [b] School of Science, Technology and Engineering, University of the Sunshine Coast, 4 Locked Bag, Maroochydore DC, Queensland 4558, Australia

[c] Institute for Marine and Antarctic Studies, University of Tasmania, Private Bag 49, Hobart, Tasmania 7001, Australia

[d] School of Biotechnology, International University, Vietnam National University, 700000 Ho Chi Minh City, Vietnam

***** Corresponding authors: Prof Abigail Elizur (aelizur@usc.edu.au) and A/Prof Tomer Ventura (tventura@usc.edu.au), Thu Thi Minh Vo (thu.vo@research.usc.edu.au)

Supplementary File 3. The expression of key DEGs in HN and HB fish when comparing the BC and FD regions within same phenotype. Downregulated genes are highlighted in gray shading.

Gene name	Log 2-Fold Change	
	HN fish	HB fish
Extracellular matrix		
collagen alpha-2(VIII) chain-like *(cola2viii)*	-2.00	-
collagen alpha-1(X) chain-like *(cola1x)*	-7.46	-
collagen alpha-1(X) chain-like *(cola1x)*	-10.64	-
collagen alpha-1(XXI) chain-like *(cola1xxi)*	2.81	-
Calcium ion binding		
troponin T, fast skeletal muscle	-4.24	-3.51
troponin C, skeletal muscle-like	-6.17	6.46
troponin I, fast skeletal muscle	-5.85	-
parvalbumin beta	-5.36	-3.31
parvalbumin beta 2-like	-9.95	-6.46
parvalbumin 2	-5.98	-3.46
Enzymes, metabolic processes		
cathepsin M	-2.40	-2.23
lipoprotein lipase-like	-2.37	-2.33
perilipin-2	-2.26	-
