# Supplementary material for: Histological and transcriptomic analysis of muscular atrophy associated with depleted flesh pigmentation in Atlantic salmon (Salmo salar) exposed to elevated seawater temperatures
Source: Sci Rep. 2023 Mar 14;13:4218. doi: 10.1038/s41598-023-31242-2 (PMC10015013; doi:10.1038/s41598-023-31242-2)
Supplement: Supplementary file 4 — Supplementary Information 4. [file 41598_2023_31242_MOESM4_ESM.pdf]

Histological and transcriptomic analysis of muscular atrophy associated with depleted flesh pigmentation in Atlantic salmon (*Salmo salar*) exposed to elevated seawater temperatures

Thu Thi Minh Vo[a,b,d,*] thu.vo@research.usc.edu.au, Gianluca Amoroso[c] gianluca.amoroso@utas.edu.au, Tomer Ventura[a,b,*] tventura@usc.edu.au, and Abigail Elizur[a,*]aelizur@usc.edu.au

[a] Centre for Bioinnovation, [b] School of Science, Technology and Engineering, University of the Sunshine Coast, 4 Locked Bag, Maroochydore DC, Queensland 4558, Australia

[c] Institute for Marine and Antarctic Studies, University of Tasmania, Private Bag 49, Hobart, Tasmania 7001, Australia

[d] School of Biotechnology, International University, Vietnam National University, 700000 Ho Chi Minh City, Vietnam

***** Corresponding authors: Prof Abigail Elizur (aelizur@usc.edu.au) and A/Prof Tomer Ventura (tventura@usc.edu.au), Thu Thi Minh Vo (thu.vo@research.usc.edu.au)

Supplementary File 4. Differentially expressed genes in muscle tissues from BC compared to FD region.

Supplementary File 4.1 Up and down-regulated DEGs in the HN fish from BC compared to FD region.

Gene ID	Gene name	Log2 Fold Change BC-FD
Skeletal muscle collagens		
LOC106569214	collagen alpha-1(XXI) chain-like, transcript variant X1	2.81
LOC106588826	collagen alpha-2(VIII) chain-like	-2.01
LOC106571324	collagen alpha-1(X) chain-like	-7.46
LOC106607727	collagen alpha-1(X) chain-like	-10.64
Muscle fiber composition protein network		
LOC106599901	myosin-6-like	9.64
LOC106569818	myosin-13-like	9.13
LOC106569817	myosin heavy chain, fast skeletal muscle-like	8.29
LOC106593049	myosin heavy chain, fast skeletal muscle-like	7.82
LOC106600226	myosin heavy chain, fast skeletal muscle-like	7.73
LOC106569812	myosin heavy chain, fast skeletal muscle-like	6.65
LOC106598369	myosin heavy chain, fast skeletal muscle-like	6.26
LOC106597657	myosin heavy chain, fast skeletal muscle-like	5.13
LOC106564183	myosin heavy chain, fast skeletal muscle-like	4.22
LOC106598781	myosin-3-like	4.1
LOC106593162	myosin heavy chain, fast skeletal muscle-like	4.07
LOC106593168	myosin heavy chain, fast skeletal muscle-like	2.92
LOC106566023	myosin-binding protein H-like, transcript variant X2	2.88
LOC106563728	myozenin-2-like, transcript variant X3	2.15
LOC106584678	unconventional myosin-IXa-like, transcript variant X1	-2.2
LOC106606262	myosin heavy chain, fast skeletal muscle-like	-2.23
LOC106564163	myosin heavy chain, fast skeletal muscle-like	-2.34
LOC106596578	myosin heavy chain, fast skeletal muscle-like	-2.74
LOC106596216	myosin heavy chain, fast skeletal muscle-like	-2.38
LOC106600781	myosin heavy chain, fast skeletal muscle-like	-3.18
LOC106593408	myosin heavy chain, fast skeletal muscle-like	-3.34
LOC106599733	myosin heavy chain, fast skeletal muscle-like	-3.4
LOC106586085	myosin heavy chain, fast skeletal muscle-like	-3.95
LOC106589415	myozenin-1-like, transcript variant X4	-4.72
LOC106591213	myosin heavy chain, fast skeletal muscle-like	-5.2
LOC106601538	myosin-binding protein C, fast-type-like, transcript variant X27	-6
LOC106598139	myosin heavy chain, fast skeletal muscle-like	-6.24
LOC106606346	myosin heavy chain, fast skeletal muscle-like	-6.99

LOC106598177	myosin-binding protein C, slow-type-like	-8.05
LOC106606178	myosin heavy chain, fast skeletal muscle-like	-8.49
LOC106598662	myosin heavy chain, fast skeletal muscle-like	-8.53
mypc1	Myosin-binding protein C, slow-type	-8.83
LOC106592374	myosin-binding protein C, fast-type-like	-9.02
LOC106596646	myosin heavy chain, fast skeletal muscle-like	-9.66
LOC106593601	myosin-4-like	-10.3
LOC106600328	myosin heavy chain, fast skeletal muscle-like	-10.54
LOC106564176	myosin heavy chain, fast skeletal muscle-like	-10.62
Calcium ion binding		
tnni3	Troponin I, cardiac muscle	8.02
prvt	Parvalbumin, thymic	-3.79
LOC106607439	parvalbumin-7-like, transcript variant X1	-3.81
LOC106609864	parvalbumin beta 3-like	-4.97
LOC106591443	parvalbumin alpha	-4.98
prvb	parvalbumin beta	-5.36
pvalb2	parvalbumin 2	-5.98
LOC106609875	parvalbumin beta 2-like	-9.95
LOC106561017	troponin I, fast skeletal muscle-like	-2.63
LOC106572105	troponin C, skeletal muscle	-3.15
tnni2	troponin I type 2 (skeletal, fast)	-3.63
LOC106584520	troponin I, fast skeletal muscle-like	-3.84
LOC106587935	troponin T, fast skeletal muscle isoforms-like	-4.24
LOC106587936	troponin T, fast skeletal muscle isoforms-like	-4.92
LOC106561014	troponin I, fast skeletal muscle-like	-6.13
LOC106566456	troponin C, skeletal muscle-like	-6.17
LOC106602975	endosialin-like	-2.17
LOC106608042	otoferlin-like	-3.27
LOC106608069	otoferlin-like	-3.66
Transporters		
slc5a12	solute carrier family 5 (sodium/monocarboxylate cotransporter), member 12	3.22
slc7a8	solute carrier family 7 (amino acid transporter light chain, L system), member 8, transcript variant X2	2.46
Enzymes, metabolic processes		
LOC106612793	dual specificity protein phosphatase 14-like, transcript variant X2	5.76
LOC106588290	serine/threonine-protein kinase SBK1-like	4.18
LOC106603872	stanniocalcin-2-like	3.52
LOC106567104	6-phosphofructo-2-kinase/fructose-2,6-bisphosphatase-like, transcript variant X3	2.67
LOC106567105	inter-alpha-trypsin inhibitor heavy chain H6-like, transcript variant X3	2.66

LOC106580788	large neutral amino acids transporter small subunit 4-like, transcript variant X2	2.61
ankmy1	ankyrin repeat and MYND domain containing 1, transcript variant X3	2.41
LOC106603085	type II iodothyronine deiodinase-like	2.39
LOC106603946	serine/arginine repetitive matrix protein 3-like	2.27
LOC106570167	serine/threonine-protein kinase SBK1-like	2.27
LOC106584023	low-density lipoprotein receptor-related protein 8-like, transcript variant X2	2.24
LOC106612881	serine/threonine-protein kinase PAK 1-like, transcript variant X1	2.21
LOC106593909	protein-lysine methyltransferase METTL21C-like	2.15
LOC106585473	protein phosphatase PTC7 homolog	2.14
LOC106572850	monocarboxylate transporter 5-like, transcript variant X2	2.1
LOC100194587	protein phosphatase 1, regulatory (inhibitor) subunit 1b-like	2.1
LOC106577817	thioredoxin-like	-2.1
aebp1	AE binding protein 1, transcript variant X1	-2.11
LOC106587252	haptoglobin-like	-2.12
LOC106584075	dual specificity protein phosphatase 22-B-like	-2.19
LOC106600514	glutamyl aminopeptidase-like	-2.19
LOC106578451	choline-phosphate cytidylyltransferase B-like, transcript variant X2	-2.24
fbln1	fibulin 1	-2.25
LOC106561284	glutaminase liver isoform, mitochondrial-like, transcript variant X2	-2.26
LOC106608163	thrombospondin-1-like	-2.27
LOC106588502	angiopoietin-1-like, transcript variant X1	-2.4
LOC106566600	complement component C7-like	-2.41
LOC106603367	transmembrane protease serine 13-like	-2.59
LOC106573306	diphosphoinositol polyphosphate phosphohydrolase 3-beta-like, transcript variant X2	-3.12
LOC106569794	very-long-chain enoyl-CoA reductase-like, transcript variant X4	-3.8
LOC106609189	solute carrier organic anion transporter family member 1C1-like, transcript variant X1	-4.25
LOC106584561	hyaluronidase-4-like	-4.63
LOC106600583	calcium/calmodulin-dependent protein kinase II inhibitor 2-like	-5.15
LOC106569411	angiopoietin-related protein 1-like, transcript variant X1	-6.3
LOC106613695	lipoprotein lipase-like	-2.37
catm	Cathepsin M	-2.4
Heat shock protein, Oxidative stress		
LOC106588457	interleukin-11-like	3.55

LOC106565287	probable flavin-containing monoamine oxidase A, transcript variant X7	-2.03
aifm2	apoptosis-inducing factor, mitochondrion-associated, 2, transcript variant X2	-2.47

Supplementary File 4.2. Up and down-regulated DEGs in the HB fish from BC compared to FD region.

Gene ID	Gene name	Log2 Fold Change BC-FD
Muscle fiber composition protein network		
LOC106569817	myosin heavy chain, fast skeletal muscle-like	6.35
LOC106593049	myosin heavy chain, fast skeletal muscle-like	6
LOC106599901	myosin-6-like	5.87
LOC106569812	myosin heavy chain, fast skeletal muscle-like	5.58
LOC106569818	myosin-13-like	5.26
LOC106569813	myosin-7-like	5.17
LOC106593162	myosin heavy chain, fast skeletal muscle-like	2.42
LOC106564176	myosin heavy chain, fast skeletal muscle-like	-8.19
LOC106593601	myosin-4-like	-8.02
LOC106598662	myosin heavy chain, fast skeletal muscle-like	-7.68
LOC106606346	myosin heavy chain, fast skeletal muscle-like	-5.86
LOC106598139	myosin heavy chain, fast skeletal muscle-like	-5.03
LOC106591213	myosin heavy chain, fast skeletal muscle-like	-4.55
mypc1	Myosin-binding protein C, slow-type	-3.07
Calcium ion binding		
tnni3	Troponin I, cardiac muscle	6.75
LOC106587935	troponin T, fast skeletal muscle isoforms-like	-3.51
LOC106609875	parvalbumin beta 2-like	-6.46
pvalb2	parvalbumin 2	-3.46
prvb	parvalbumin beta	-3.31
LOC106566456	troponin C, skeletal muscle-like	-2.21
LOC106584318	voltage-dependent R-type calcium channel subunit alpha-1E-like, transcript variant X5	-2.67
Lipid and fatty acid metabolism		
LOC106582767	retinoid-binding protein 7-like	-2.23
LOC106613695	lipoprotein lipase-like	-2.33
Enzymes, metabolic processes		
LOC106612793	dual specificity protein phosphatase 14-like, transcript variant X2	5.43
LOC106571490	serine protease 23-like	2.66
LOC106582920	inositol hexakisphosphate kinase 2-like	2.63
LOC106570167	serine/threonine-protein kinase SBK1-like	2.19
LOC106610249	monocarboxylate transporter 4-like, transcript variant X2	2.12
LOC106603085	type II iodothyronine deiodinase-like	2.68
LOC106570167	serine/threonine-protein kinase SBK1-like	2.19
LOC106610249	monocarboxylate transporter 4-like, transcript variant X2	2.12
LOC106603085	type II iodothyronine deiodinase-like	2.68

pappa2	pappalysin 2, transcript variant X1	-2.48
catm	Cathepsin M	-2.23
LOC106609416	PRKC apoptosis WT1 regulator protein-like, transcript variant X2	-2.13
