# Supplementary material for: Histological and transcriptomic analysis of muscular atrophy associated with depleted flesh pigmentation in Atlantic salmon (Salmo salar) exposed to elevated seawater temperatures
Source: Sci Rep. 2023 Mar 14;13:4218. doi: 10.1038/s41598-023-31242-2 (PMC10015013; doi:10.1038/s41598-023-31242-2)
Supplement: Supplementary file 5 — Supplementary Information 5. [file 41598_2023_31242_MOESM5_ESM.pdf]

Histological and transcriptomic analysis of muscular atrophy associated with depleted flesh pigmentation in Atlantic salmon (*Salmo salar*) exposed to elevated seawater temperatures

Thu Thi Minh Vo[a,b,d,*] thu.vo@research.usc.edu.au, Gianluca Amoroso[c] gianluca.amoroso@utas.edu.au, Tomer Ventura[a,b,*] tventura@usc.edu.au, and Abigail Elizur[a,*]aelizur@usc.edu.au

[a] Centre for Bioinnovation, [b] School of Science, Technology and Engineering, University of the Sunshine Coast, 4 Locked Bag, Maroochydore DC, Queensland 4558, Australia

[c] Institute for Marine and Antarctic Studies, University of Tasmania, Private Bag 49, Hobart, Tasmania 7001, Australia

[d] School of Biotechnology, International University, Vietnam National University, 700000 Ho Chi Minh City, Vietnam

***** Corresponding authors: Prof Abigail Elizur (aelizur@usc.edu.au) and A/Prof Tomer Ventura (tventura@usc.edu.au), Thu Thi Minh Vo (thu.vo@research.usc.edu.au)

Supplementary File 5. Primers designed for validation of *in silico* expression values determined by RNA-Seq analysis

Gene	Forward primer	Reverse primer
collagen alpha-1(I) chain *(cola1i)*	gattcgacatgggcttcatt	gaagtgacggaagggatcg
collagen alpha-1(XI) chain *(cola1xi)*	aacgtccgccagtacaagat	gagacctgggacacatcctc
collagen alpha-1(XII) chain *(cola1xii)*	ggtgagatcggacaacgtg	agggccaagcagacctcta
leptin receptor *(lepr)*	taacagccaacgtgtccaac	gcggagggacaatgagaat
ATP-dependent 6-phosphofructokinase, muscle type *(atpd6p)*	acggaaaccccattagctct	agtggtacgggtgtcaaagc
troponin C, skeletal muscle *(tnc)*	ttcctttgtgaccctttcca	ggcttaaattttgtatttgattttcc
apolipoprotein C-I *(apoci)*	agatgaaggccaagatcgac	tagacaggcagggtctgagg
apolipoprotein Eb *(apoeb)*	gtgtggagccctttgtgagt	Gttcttcagaaggtcagtgaagc
actin, alpha skeletal muscle 2-like (*acta2*)	gtaacgagcgtttccgttg	ggcagactccataccaatgaa
tropomyosin beta chain (*tpmsb*)	cttcctcctcctcctcctcc	tggaggatgtctgaacccca
thymosin beta (*thymb*)	tgaagaggacgtgaggagga	aaacaaagtgcttttgttgacg
myostatin 1b (*myo1b*)	gcagatcatctacggcaagat	agcagaactcccgctcac
lipoprotein lipase-like (*lipase*)	ggtgacctcctgatggtaaagt	atggatgaggctgaccagtt
fatty acid-binding protein, intestinal-like (*fabpi*)	ccacatcagcagacagataatca	ccgtttgcctcccttttat
cathepsin L1-like (*catl*)	gcattccgtttatttgtaacttttc	aactgacatgttatgtgctagagactg
lipase, hormone-sensitive (*lipe*)	cattggttacaacgccaaga	cagcacgggtcttcaggta
calsequestrin-like (*calse*)	agatgacttcccgctgctt	tggggagagcctaggtcaat
heat shock 70 kDa protein 4 (*hsp70*)	agatcgttgaagcttataaaaccaa	cgccatctgacaatggtgt
