# Supplementary material for: Histological and transcriptomic analysis of muscular atrophy associated with depleted flesh pigmentation in Atlantic salmon (Salmo salar) exposed to elevated seawater temperatures
Source: Sci Rep. 2023 Mar 14;13:4218. doi: 10.1038/s41598-023-31242-2 (PMC10015013; doi:10.1038/s41598-023-31242-2)
Supplement: Supplementary file 6 — Supplementary Information 6. [file 41598_2023_31242_MOESM6_ESM.pdf]

Histological and transcriptomic analysis of muscular atrophy associated with depleted flesh pigmentation in Atlantic salmon (*Salmo salar*) exposed to elevated seawater temperatures

Thu Thi Minh Vo[a,b,d,*] thu.vo@research.usc.edu.au, Gianluca Amoroso[c] gianluca.amoroso@utas.edu.au, Tomer Ventura[a,b,*] tventura@usc.edu.au, and Abigail Elizur[a,*]aelizur@usc.edu.au

[a] Centre for Bioinnovation, [b] School of Science, Technology and Engineering, University of the Sunshine Coast, 4 Locked Bag, Maroochydore DC, Queensland 4558, Australia

[c] Institute for Marine and Antarctic Studies, University of Tasmania, Private Bag 49, Hobart, Tasmania 7001, Australia

[d] School of Biotechnology, International University, Vietnam National University, 700000 Ho Chi Minh City, Vietnam

***** Corresponding authors: Prof Abigail Elizur (aelizur@usc.edu.au) and A/Prof Tomer Ventura (tventura@usc.edu.au), Thu Thi Minh Vo (thu.vo@research.usc.edu.au)

Supplementary File 6. Statistical analysis of qPCR in the BC region.

Statistics.R

ttmvo

2023-01-02

```
library("ggplot2")
```

```
## Warning: package 'ggplot2' was built under R version 4.2.2
```

```
library("ggpubr")

###BC region
setwd("C:/Users/ttmvo/Dropbox/Atlantic salmon/New RNAseq/NewThresholdAnalysis/")
directory <- "C:/Users/ttmvo/Dropbox/Atlantic salmon/New RNAseq/NewThresholdAnalysis/"

a <- read.delim(file = "BC.txt", header = TRUE, sep = "\t")


kruskal.test(acta2 ~ Group, data = a)
```

```
##
##  Kruskal-Wallis rank sum test
##
## data:  acta2 by Group
## Kruskal-Wallis chi-squared = 5.18, df = 2, p-value = 0.07502
```

```
pairwise.wilcox.test(a$acta2, a$Group,
                     p.adjust.method = "fdr")
```

```
##
##  Pairwise comparisons using Wilcoxon rank sum exact test
##
## data:  a$acta2 and a$Group
##
##    HB    HN
## HN 0.226 -
## P  0.690 0.095
##
## P value adjustment method: fdr
```

```
kruskal.test(thymb ~ Group, data = a)
```

```
##
##  Kruskal-Wallis rank sum test
##
## data:  thymb by Group
## Kruskal-Wallis chi-squared = 7.34, df = 2, p-value = 0.02548
```

```
pairwise.wilcox.test(a$thymb, a$Group,
                     p.adjust.method = "fdr")
```

```
##
##  Pairwise comparisons using Wilcoxon rank sum exact test
##
## data:  a$thymb and a$Group
##
##    HB    HN
## HN 0.841 -
## P  0.048 0.048
##
## P value adjustment method: fdr
```

```
kruskal.test(tpmsnb ~ Group, data = a)
```

```
##
##  Kruskal-Wallis rank sum test
##
## data:  tpmsnb by Group
## Kruskal-Wallis chi-squared = 5.66, df = 2, p-value = 0.05901
```

```
pairwise.wilcox.test(a$tpmsnb, a$Group,
                     p.adjust.method = "fdr")
```

```
##
##  Pairwise comparisons using Wilcoxon rank sum exact test
##
## data:  a$tpmsnb and a$Group
##
##    HB    HN
## HN 0.690 -
## P  0.143 0.095
##
## P value adjustment method: fdr
```

```
kruskal.test(calse ~ Group, data = a)
```

```
##
##  Kruskal-Wallis rank sum test
##
## data:  calse by Group
## Kruskal-Wallis chi-squared = 6.72, df = 2, p-value = 0.03474
```

```
pairwise.wilcox.test(a$calse, a$Group,
                     p.adjust.method = "fdr")
```

```
##
##  Pairwise comparisons using Wilcoxon rank sum exact test
##
## data:  a$calse and a$Group
##
##    HB    HN
## HN 0.310 -
## P  0.083 0.083
##
## P value adjustment method: fdr
```

```
kruskal.test(fabi ~ Group, data = a)
```

```
##
##  Kruskal-Wallis rank sum test
##
## data:  fabi by Group
## Kruskal-Wallis chi-squared = 10.5, df = 2, p-value = 0.005248
```

```
pairwise.wilcox.test(a$fabi, a$Group,
                     p.adjust.method = "fdr")
```

```
##
##  Pairwise comparisons using Wilcoxon rank sum exact test
##
## data:  a$fabi and a$Group
##
##    HB    HN
## HN 0.151 -
## P  0.012 0.012
##
## P value adjustment method: fdr
```

```
kruskal.test(lipe ~ Group, data = a)
```

```
##
##  Kruskal-Wallis rank sum test
##
## data:  lipe by Group
## Kruskal-Wallis chi-squared = 7.44, df = 2, p-value = 0.02423
```

```
pairwise.wilcox.test(a$lipe, a$Group,
                     p.adjust.method = "fdr")
```

```
##
##  Pairwise comparisons using Wilcoxon rank sum exact test
##
## data:  a$lipe and a$Group
##
##    HB    HN
## HN 0.024 -
## P  0.083 0.841
##
## P value adjustment method: fdr
```

```
kruskal.test(lipase ~ Group, data = a)
```

```
##
##  Kruskal-Wallis rank sum test
##
## data:  lipase by Group
## Kruskal-Wallis chi-squared = 6.74, df = 2, p-value = 0.03439
```

```
pairwise.wilcox.test(a$lipase, a$Group,
                     p.adjust.method = "fdr")
```

```
##
##  Pairwise comparisons using Wilcoxon rank sum exact test
##
## data:  a$lipase and a$Group
##
##    HB    HN
## HN 0.841 -
## P  0.083 0.048
##
## P value adjustment method: fdr
```

```
wilcox.test(a$myo1b ~ a$Group)
```

```
##
##  Wilcoxon rank sum exact test
##
## data:  a$myo1b by a$Group
## W = 20, p-value = 0.1508
## alternative hypothesis: true location shift is not equal to 0
```

```
wilcox.test(a$catl ~ a$Group)
```

```
##
##  Wilcoxon rank sum exact test
##
## data:  a$catl by a$Group
## W = 2, p-value = 0.03175
## alternative hypothesis: true location shift is not equal to 0
```

```
wilcox.test(a$hsp70 ~ a$Group)
```

```
##
##  Wilcoxon rank sum exact test
##
## data:  a$hsp70 by a$Group
## W = 20, p-value = 0.1508
## alternative hypothesis: true location shift is not equal to 0
```
