# Supplementary material for: Histological and transcriptomic analysis of muscular atrophy associated with depleted flesh pigmentation in Atlantic salmon (Salmo salar) exposed to elevated seawater temperatures
Source: Sci Rep. 2023 Mar 14;13:4218. doi: 10.1038/s41598-023-31242-2 (PMC10015013; doi:10.1038/s41598-023-31242-2)
Supplement: Supplementary file 7 — Supplementary Information 7. [file 41598_2023_31242_MOESM7_ESM.pdf]

Histological and transcriptomic analysis of muscular atrophy associated with depleted flesh pigmentation in Atlantic salmon (*Salmo salar*) exposed to elevated seawater temperatures

Thu Thi Minh Vo[a,b,d,*] thu.vo@research.usc.edu.au, Gianluca Amoroso[c] gianluca.amoroso@utas.edu.au, Tomer Ventura[a,b,*] tventura@usc.edu.au, and Abigail Elizur[a,*]aelizur@usc.edu.au

[a] Centre for Bioinnovation, [b] School of Science, Technology and Engineering, University of the Sunshine Coast, 4 Locked Bag, Maroochydore DC, Queensland 4558, Australia

[c] Institute for Marine and Antarctic Studies, University of Tasmania, Private Bag 49, Hobart, Tasmania 7001, Australia

[d] School of Biotechnology, International University, Vietnam National University, 700000 Ho Chi Minh City, Vietnam

***** Corresponding authors: Prof Abigail Elizur (aelizur@usc.edu.au) and A/Prof Tomer Ventura (tventura@usc.edu.au), Thu Thi Minh Vo (thu.vo@research.usc.edu.au)

Supplementary File 7. The classification of flesh phenotypes based on SalmonFan^{TM} score

Front dorsal region	Back central region	Flesh color phenotype
≥ 25	≥ 25	High color-no banding fish
≤ 23	> 25	High color-banded fish
≤ 23	≤ 23	Pale fish
